# Phosphorylation of the proline-rich domain of WAVE3 drives its oncogenic activity in breast cancer

**DOI:** 10.1038/s41598-021-83479-4

**Published:** 2021-02-16

**Authors:** Urna Kansakar, Wei Wang, Vesna Markovic, Khalid Sossey-Alaoui

**Affiliations:** 1 Department of Medicine, Rammelkamp Center for Research, MetroHealth, Cleveland, OH USA; 2 Case Western Reserve University School of Medicine, Cleveland, OH USA; 3 Case Comprehensive Cancer Center, Cleveland, OH USA; 4 Department of Medicine, Case Western Reserve University School of Medicine, Rammelkamp Center for Research, R457, 2500 MetroHealth Drive, Cleveland, OH 44109 USA

**Keywords:** Biological techniques, Cancer, Cell biology, Molecular biology, Stem cells, Cardiology, Molecular medicine, Oncology, Pathogenesis, Diseases, Cancer

## Abstract

Post-translational modification of proteins, such as tyrosine phosphorylation, plays a major role in driving the oncogenic activity of oncogenes. WAVE3 (WASF3), an adaptor and actin cytoskeleton remodeling protein, contributes to cell migration, cancer cell invasion, and metastasis. WAVE3 plays a vital role in the progression and metastasis of triple negative breast cancer (TNBC), in part through the regulation of cancer stem cells (CSCs). Several studies have shown that WAVE3 tyrosine phosphorylation is required for its oncogenic activity. Moreover, our recent study showed that the proline rich domain (PRD) of WAVE3 is required for maintenance of the CSC niche in breast cancer by regulating the nuclear translocation of the CSC-specific nuclear transcription factor YB1. Here, we show that the PRD domain of WAVE3 and its phosphorylation are essential for driving the oncogenic activity of WAVE3. We show that phosphorylation of WAVE3 PRD is essential for migration and invasion of breast cancer cells in vitro, as well as tumor growth and metastasis in vivo. Mechanistically, we show that phosphorylation of the WAVE3 PRD is essential for interaction between WAVE3 and YB1. Loss of PRD phosphorylation inhibits such interaction and the YB1-mediated activation of expression of CSC markers, as well as the WAVE3 mediated activation of EMT. Together, our study identifies a novel role of WAVE3 and its PRD domain in the regulation of the invasion metastasis cascade in BC that is independent of the known function of WAVE3 as an actin cytoskeleton remodeling protein through the WAVE regulatory complex (WRC).

## Introduction

Cancer metastasis is a complex process that allows tumor cells to escape the primary site and colonize distant organs via the blood circulation or the lymphatic system^1^. Metastasis also accounts for more than 90% of cancer-related deaths^2^. WAVE3 (WASF3) is a member of the Wiskott-Aldrich Syndrome Protein (WASP) family that was originally identified as regulator of the actin cytoskeleton remodeling^3,4^. Several other studies have also implicated WAVE3 in several aspects of cell migration, cancer cell invasion and tumor metastasis (reviewed in Refs.^3,5^. WAVE3, consists of several functional domains: (i) an N-terminal basic region (BR), (ii) a central proline-rich domain (PRD) which is known to play an important role for the enhancement of Cdc42-WASP-induced nucleation of actin polymerization, and (iii) a C-terminal Verprolin homology, Cofilin homology and Acidic (VCA) domain, that binds to the Arp2/3 complex and initiates the growth of actin filaments required for cell motility and migration^6–8^. Like WAVE1 (WASF1) and WAVE2 (WASF2), the function of WAVE3 in actin polymerization and actin cytoskeleton remodeling is regulated through its inclusion in the WAVE regulatory complex (WRC)^9^. WRC consists of five protein subunits: PIR121, Nap125, HSPC300, and Abi1, where WAVE3 is kept in an inactive state through the formation of a complex with the other four protein subunits of the WRC^9,10^. Subsequent recruitment of Rho GTPase Rac1 to the WRC releases WAVE3 from this complex^11^, which becomes free to bind to the actin nucleator Arp2/3 complex and activate actin polymerization^9,12,13^. Recent studies from our group have however identified a function for WAVE3 that may be independent of its traditional relationship with the WRC, Arp2/3 complex and actin polymerization^14,15^.

Post-translational modifications (PTMs) of proteins plays a major role in driving the oncogenic activity in several cancer types including BC. Several studies have shown that PTM of WAVE3 through its tyrosine phosphorylation was critical for that WAVE3-mediated regulation of tumor progression and metastasis of TNBC^15–17^. Phosphorylation of WAVE3 at four tyrosine residues (Y151, Y248, Y337, and Y486) is mediated by the non-receptor tyrosine kinase c-Abl, downstream of PI3K^16^. A more recent study has shown that the oncogenic activity of WAVE3 in BC is significantly enhanced as a result of tyrosine phosphorylation^15^. Two of the four tyrosine residues of WAVE3, Y248 and Y337, reside within the PRD domain. The PRD domain of WAVE3 was also shown to be necessary for the translocation from the cytoplasm to the nucleus of the transcription factor YB1, and the subsequent activation of cancer stem cells (CSC)-specific transcription factors, such as Nanog and Oct4 and Sox^14^. This novel function of WAVE3, that is mediated by the PRD domain, seems to be independent of the WRC and actin polymerization^6,9,12,17–20^. In this study, we show that phosphorylation of WAVE3 PRD domain is required for cell migration, invasion, as well as the maintenance of the cancer stem cell phenotype in TNBC. We also show that loss of phosphorylation of WAVE3 at the PRD domain suppresses the epithelial-to-mesenchymal (EMT) programs, as well as significantly inhibits expression of CSC-specific markers through the dysregulation of the WAVE3-YB1 interaction.

Our data demonstrates that the oncogenic activity of WAVE3 can be driven by phosphorylation of its PRD domain, and targeting this PTM may serve as a potential therapeutic option for treating patients with TNBCs, thus preventing cancer progression and metastasis.

## Material and methods

### Ethics statement

All animal studies were performed under protocols approved by the Institutional Animal Care and Use Committee and conducted in accordance with the guidelines and regulations set and approved by the MetroHealth Medical Center, Case Western Reserve University, and NIH. For this study, we used six- to eight-weeks-old female NSG (Jackson Laboratory, Farmington, CT).

### Cell lines and reagents

MDA-MB-231 and 4T1 TNBC cell lines were obtained from American Type Culture Collection (ATCC). Cells were maintained in Dulbecco’s modified Eagle’s medium supplemented with 10% FBS. We used STR DNA fingerprinting analysis for regular authentication of cells that we used for this study. WAVE3-deficient (WAVE3-KO) cells were produced using lentiviral transduction as described^14,15^. For each BC cell line, we used two different and verified WAVE3-specific sgRNAs and a scrambled sgRNA i.e., nonsilencing sgRNA^14,15^. We used western blot to verify the loss of WAVE3 expression. For stimulation of cells with PDGF, cells were serum starved in serum-free DMEM without antibiotics overnight. Next day, the cells were stimulated with 100 ng/ml PDGF (Millipore) for 10 min. The PI3K inhibitor LY294002 was obtained from SelleckChem and used at a concentration of 10 µM for 4 h.

### Antibodies and other reagents

Rabbit anti-WAVE3, rabbit anti-YB1, mouse anti-phosphotyrosine (PY20) primary antibodies were obtained from Cell Signaling Technology (CST) and used at a dilution of 1:1,000. Mouse anti-GFP was obtained from Clontech Laboratories, Inc. and used at a dilution of 1:5000. Goat horseradish peroxidase-conjugated anti-mouse and anti-rabbit IgG were purchased from Calbiochem, Sigma-Aldrich and used at a dilution of 1:2000. Mouse monoclonal anti-β-Actin was obtained from Sigma-Aldrich and used at a dilution of 1:5000. Mouse anti-E-cadherin antibody (1:200) was from BD Transduction Laboratories and mouse anti-Vimentin antibody (1:200) was from Santa Cruz Biotechnology, Inc. Alexa Fluor 594-conjugated donkey anti-mouse IgG (1:500) and Hoechst 33,342 (1:1000) were from Invitrogen. Gel electrophoresis reagents were obtained from Bio-Rad. All the qRT-PCR primers were obtained from Integrated DNA Technologies (IDT) and are listed in Table 1.

**Table 1:** List of primers and their sequences used in this study.

Cloning and mutagenesis primers	
W3-PRD-F	5′-GCA AAG AAA CTG GAG CAG GC-3′
W3-PRD-R	5′-TTA GGG GCC ATG CAT TGG GGA-3′
WAVE3Y151F	5′-TGAAGTTCTtTACTGATCCTTCC-3′
WAVE3Y151R	5′-GGAAGGATCAGTAaAGAACTTCA-3′
WAVE3Y486F	5′-GCCGTGGAGTtCAGCGACTCT-3′
WAVE3Y486R	5′-AGAGTCGCTGaACTCCACGGC-3′
WAVE3Y248F	5′-ACGGATTtCTCTTACCCGGCT-3′
WAVE3Y248R	5′-AGCCGGGTAAGAGaAATCCGT-3′
WAVE3Y337F	5′-GATAATTGAGTtTTACAACCCA-3′
WAVE3Y337R	5′-TGGGTTGTAAaACTCAATTATC-3′
**Quantitative RT-PCR primers**	
Oct4-F	5′-CACATGAAGGAGCACCCGGATTAT-3′
Oct4-R	5′-AACTTCACCTTCCCTCCAACCAGT-3′
Nanog-F	5′-CCCAAAGGCAAACAACCCACTTCT-3′
Nanog-R	5′-AGCTGGGTGGAAGAGAACACAGTT-3′
E-cadherin-F	5′-GGCACAGATGGTGTGATTACAGTCAAAA-3′
E-cadherin-R	5′-GTCCCAGGCGTAGACCAAGAAA-3′
N-cadherin-F	5′-GACGGTTCGCCATCCAGAC-3′
N-cadherin-R	5′-TCGATTGGTTTGACCACGG-3′
Zeb1-F	5′-GCCAATAAGCAAACGATTCTG-3′
Zeb1-R	5′-TTTGGCTGGATCACTTTCAAG-3′
Zeb2-F	5′-GGTCCAGATCGAAGCAGCTCAAT-3′
Zeb2-R	5′-GTGACTTCTATGTTTGTTCACATT-3′
Twist-F	5′-GGACAAGCTGAGCAAGATTCAGA-3′
Twist-R	5′-TCTGGAGGACCTGGTAGAGGAA-3′
Vimentin-F	5′-CCAGGCAAAGCAGGAGTC-3′
Vimentin-R	5′-CGAAGGTGACGAGCCATT-3′
Snail-F	5′-CCCCAATCGGAAGCCTAA-3′
Snail-R	5′-CCTTTCCCACTGTCCTCAT-3′
TGF-β-F	5′-CCCTGGCTGTCCTTATCATC-3′
TGF-β-R	5′-ACTGTTTCTGAGTGGCAGCA-3′
GAPDH-F	5′-GGATTTGGTCGTATTGGG-3′
GAPDH-R	5′-GGAAGATGGTGATGGGATT-3′
**Genomic DNA PCR primers**	
Hu-ERVK6-F	5′-AGGGACTAGGGAAAAATGAAGATG-3′
Hu-ERVK6-R	5′-GGGTTGAATTACGGCGTTTACAGC-3′
Mo-FERMT2-F	5′-CTTCCCTCAGTGATGGAGTGTGATCTGAG-3′
Mo-FERMT2-R	5′-GGAGTCAGAGAGAATGGGCACTCTAGGTG-3′

### Immunoblotting

Immunoblotting analyses were performed as previously described^21,22^. Cells were grown in 6 well plates and washed twice with ice-cold PBS. Immediately, ice-cold RIPA lysis buffer in the presence of a mixture of proteases and phosphatase inhibitors. Cells were rapidly scraped off the plates, and the crude lysates were transferred to prechilled tubes and centrifuged at 15,000 RPM for 20 min at 4 °C. For immunoblotting, cell lysates containing equivalent amounts of total protein (25 μg) were supplemented with SDS sample buffer and resolved on a 10% SDS–polyacrylamide gel, followed by transfer to PVDF membranes using Bio-Rad gel and transfer apparatus. Membranes were incubated in 5% bovine serum albumin (BSA) for 1 h at room temperature, washed with PBS, followed by incubation with the primary antibody overnight at 4 °C. Membranes were then washed and incubated in the appropriate secondary antibody at room temperature for 1 h, and the signals were developed using a Pierce ECL Western blot chemiluminescence detection kit and visualized with ChemiDoc MP Imaging System. For signal quantification, ImageJ software was used according to the parameters described in ImageJ user guide (http://rsbweb.nih.gov/ij/docs/guige/146.html). Average values from 3 different blots are presented.

### Cell growth and migration assays

Cell proliferation was also assessed by counting the number of viable cells. Cells (2.5 × 10^4^) were seeded into six well-plates in complete growth medium, then harvested at one day intervals over 5 days, and counted using a hemocytometer as previously described^23–25^. Cell viability was assessed using trypan blue staining. For wound-healing assay, cells were grown to confluency and a scratch was made by dragging a thin pipet tip across the cell surface. Cells were washed with serum-free medium to remove detached cells and then incubated in IncuCyte Live-Cell Analysis System. Images were taken every 24 h using the same system. Wound closure was quantified by measuring the width of the remaining wound using ImageJ 1.8.0 (NIH). Assays were performed in triplicates, and the values plotted were the average of three independent experiments.

### Three-dimensional tumorsphere cultures

#### 3D spheroid invasion

MDA-MB-231, 4T1 or their derivatives (1,500 cells per well) were seeded in 96-well ultra-low attachment (ULA) plate and centrifuged at 125×*g* for 10 min at room temperature. For compaction of cell aggregates into scaffold-free single spheroid formation and assessing their invasive potential, 3 days post formation of tumorspheres, each well was supplemented with 90 μl Matrigel (in complete culture media) on top. The plate was then placed inside the IncuCyte Live-Cell Analysis System and scanning was scheduled every 6 h continuously for 10 days.

### 3D multi-spheroid formation

For multi-tumorsphere formation, a 96-well ULA plate was coated with 40 μl Matrigel (in complete culture media) and placed in the incubator for 30 min to allow polymerization. Next, cells (1,500 per well) were plated on top of the layer of Matrigel and placed inside the IncuCyte Live-Cell Analysis System and scanned every 6 h continuously for 10 days to monitor multi-spheroid formation^23^.

### Immunofluorescence staining of tumorspheres

Tumorspheres, grown as described above, were transferred to a 12-well plate containing 400 µL PBS and the tumorspheres were allowed to settle in bottom of the plate for 3 min before the plate was tilted slightly and PBS removed by gentile aspiration making sure the tumorspheres are not disturbed. Tumorspheres were then fixed for 20 min at room temperature in 3.8% formalin in PBS, and permeabilized in 0.025% w/v Triton X-100 for 5 min at room temperature, followed by three washes in PBS for 3 min each. Next, tumorspheres were blocked in 5% donkey serum in PBS for 30 min and washed 3 times in PBS, then incubated overnight with the primary antibody (1:200 dilution) in 5% DS at 4 °C. Tumorspheres were carefully washed 3 times and next incubated with Alexa Fluor 594-conjugated donkey anti-mouse IgG secondary antibody (1:500 dilution) for 1 h at room temperature, followed by three washes in PBS. Nuclei were stained with Hoechst 33,342 (1:1000 dilution in PBS) for 10 min and washed 3 times. Finally, tumorspheres were observed and documented under the Leica DMi8 microscope.

### Primary tumor growth and pulmonary metastasis

In the spontaneous metastasis assay, parental (GFP), WAVE3-deficient (W3-KO), WAVE3-deficient overexpressing wild-type WAVE3-PRD-GFP fusion (PRD-WT) or phospho-mutant WAVE3-PRD-GFP fusion (PRD-DM) MDA-MB-231 cells (1 million cells per mouse, *n* = 5) were implanted into the mammary fat pads of six- to eight-weeks-old female NSG mice. Each mouse was implanted on both the left and right abdominal mammary fat pad. Mice were anaesthetized using isofluorane prior to injecting tumor cells. Tumor growth was monitored twice every week and the tumor volume was measured using Instant Readout Digital Caliper (Electron Microscopy Sciences, PA). At the endpoint, tumors were excised, weighed and fixed with 4% paraformaldehyde. For the experimental metastasis assay, cells (2 × 10^6^) suspended in 0.15 ml of sterile PBS were injected using a 28-gauge needle into a tail vein of six- to eight-weeks-old female NSG mice. Mice were sacrificed 5 weeks later, and the recovered lungs were fixed with 4% paraformaldehyde. Lung metastasis nodules were counted, and the results were plotted as average number of foci per lobe. Metastasis was also quantified by qt-PCR of human genomic DNA from total DNA of mouse lungs using human DNA specific primers as described^25^. PCR primers and their sequences are listed in Table 1.

### Expression vectors and transfections

GFP-tagged WAVE3 PRD constructs were generated as previously described^14^ and sequence-verified. GFP-tagged WAVE3 PRD was generated via ligation of the wild-type WAVE3 PRD domain (accession number AF454702) in- frame with GFP in the pEGFP-C2 expression vector (Clontech). The resulting plasmid construct was used as a template for site-directed mutagenesis to introduce the Y to F mutations using the primers listed in Table 1 as described previously^16^. The GFP-recombinant vector or the empty GFP expression control vector was used for stable transfections using standard protocols. The correct size of the fusion proteins was verified by Western blot analysis. Oligonucleotide primers used for site-directed mutagenesis and sequencing were from Qiagen and are described in Table 1 and reference^16^.

#### RNA extraction and reverse transcription PCR

RNA extraction was performed as previously described^26^. Briefly, cells were lysed in TRIzol reagent (Invitrogen), and total RNA was extracted according to the manufacturer’s instructions. Total RNA, resuspended in RNase-free water, was quantified using a Nanodrop 2000 spectrophotometer (ThermoFisher Scientific); and 1 μg of RNA was used to generate cDNA with the SuperScript III First-Strand Synthesis System RT-PCR kit (Invitrogen). The resulting cDNA was used a template for qtRT-PCR as described previously^13^, using the C1000 Touch Thermal Cycler CFX96 Real-Time System (Bio-Rad). Oligonucleotide primers used for qtRT-PCR were from Qiagen and are listed in Table 1.

### Statistical analysis

All the experiments were performed in triplicates. Data are presented as mean and S.D., and a two-tailed Student’s t-test was used to compare the samples assuming equal variances for the two populations. A *p*-value < 0.05 was considered statistically significant. For statistical analysis of qRT-PCR data, logarithmic values were converted to ΔΔCt values.

## Results

### Tyrosine phosphorylation of the PRD domain of WAVE3 in breast cancer cells is mediated by PDGF downstream of PI3K

**Figure 1:**
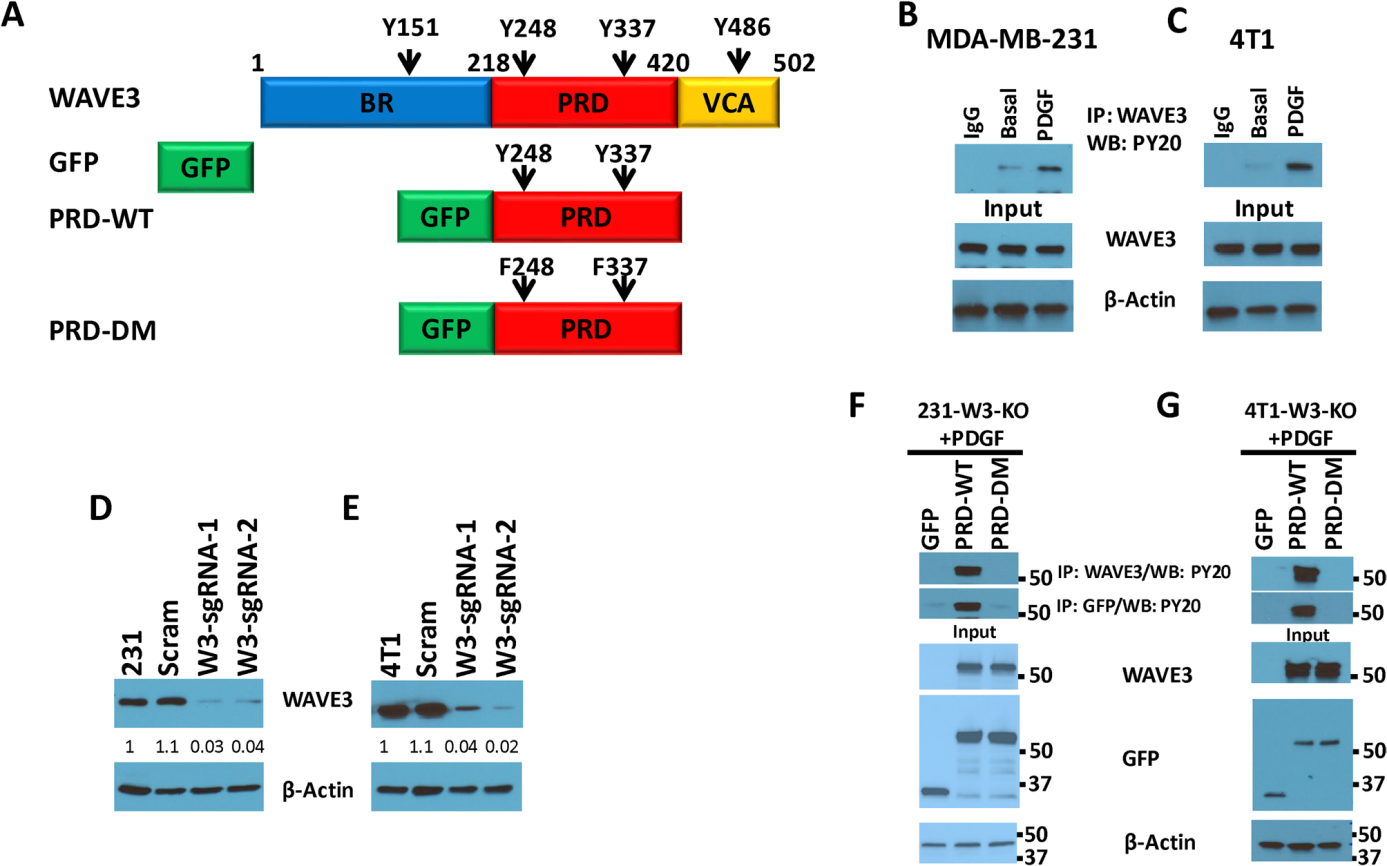
WAVE3 PRD domain is the host for two tyrosine residues the phosphorylation of which regulates regulate WAVE3 activity. (**A**) Graphical representation of WAVE3 functional domains and GFP-fused truncation mutants. Wild-type WAVE3-PRD domain (PRD-WT) is the host of two tyrosine residues (Y248 and Y337). The two tyrosine residues (Y248 and Y337) were mutated to unphosphorylated phenylalanine (F248 and F337), known as phospho-mutant (PRD-DM). (**B**,**C**) Proteins lysates prepared from MDA-MB-231 (**B**) and 4T1 (**C**) cells that treated with PDGF were used for immunoprecipitation with anti-WAVE3 antibody and subjected to immunoblotting analysis with anti-PY20 antibody. (**D**,**E**) Western blots developed with anti-WAVE3 antibody of protein lysates from MDA-MB-231 (**D**) and 4T1 (**E**) cells transduced with a scrambled sgRNA (Scram), WAVE3 sgRNA-1 (W3-sgRNA-1), WAVE3 sgRNA-2 (W3-sgRNA-2). β-Actin is a loading control. (**F**,**G**) Protein lysates prepared from PDGF-treated WAVE3-deficient MDA-MB-231 (**F**) and 4T1 (**G**) cells that were transfected with GFP, wild-type WAVE3-PRD-GFP fusion (PRD-WT) or phospho-mutant WAVE3-PRD-GFP fusion (PRD-DM), were used for immunoprecipitation with anti-WAVE3 antibody and subjected to immunoblotting analysis with anti-PY20 antibody. Cell lysates were also immunoblotted with anti-WAVE3 and anti-GFP antibodies to show expression of the fusion proteins and the presence of equal amounts of these proteins in the cell lysates (input panels). β-Actin is a loading control.

**Figure 2:**
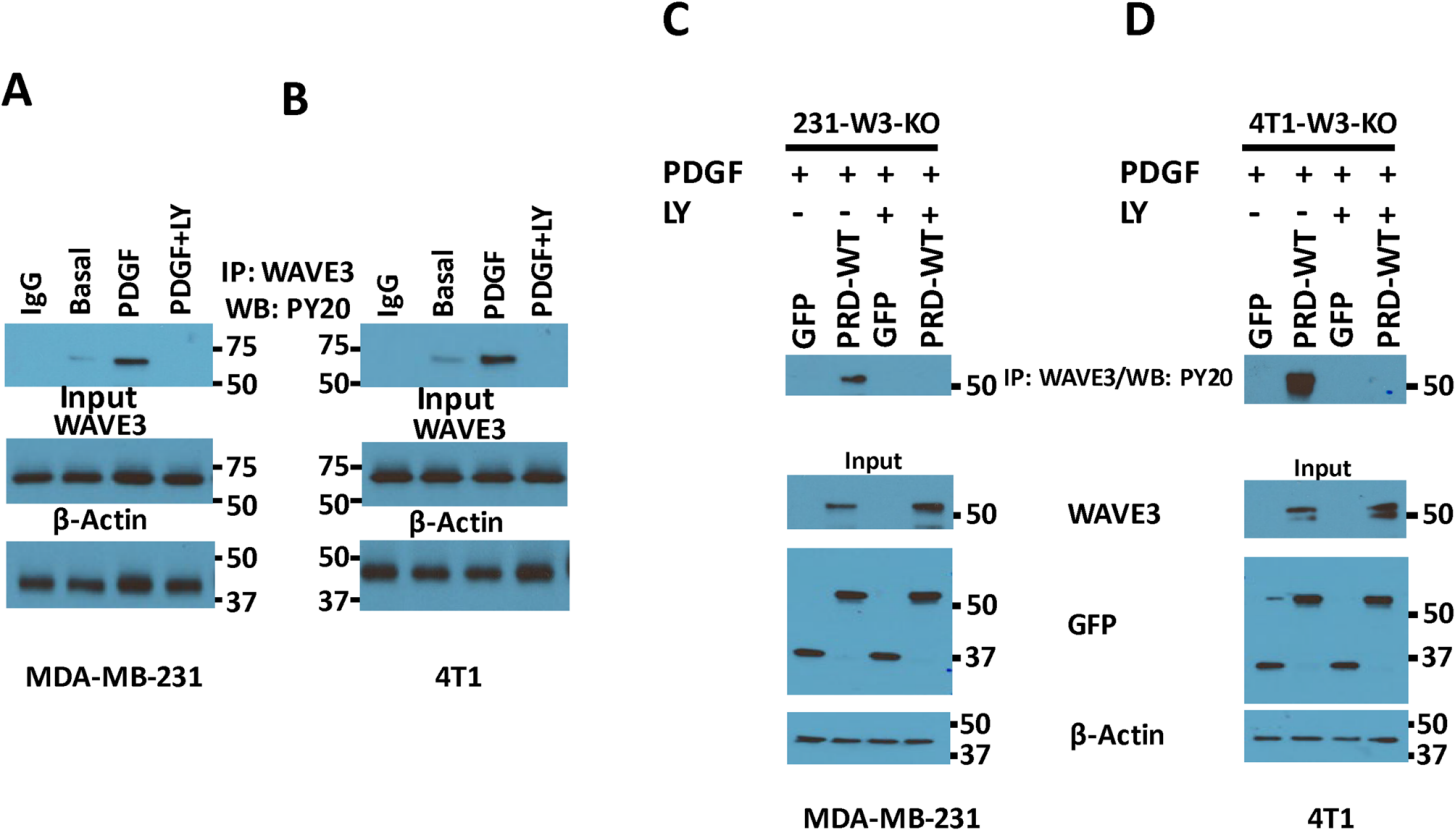
WAVE3 PRD tyrosine phosphorylation in breast cancer cells is mediated by PDGF. (**A**,**B**) Protein lysates of MDA-MB-231 (**A**) and 4T1 (**B**) cells that were treated with either PDGF or with both PDGF and LY were immunoprecipitated with anti-WAVE3 antibody and subjected to immunoblotting with anti-phospho-tyrosine PY20. Rabbit IgG was used as a negative control (top panels). The presence of equal amounts of WAVE3 and proteins in the cell lysates was confirmed by immunoblotting with anti-WAVE3 (Input, middle panel). (**C**,**D**) Cell lysates prepared from WAVE3-deficient MDA-MB-231 (**C**) and 4T1 (**D**) cells that were transfected with GFP, wild-type WAVE3-PRD-GFP fusion (PRD-WT) or phospho-mutant WAVE3-PRD-GFP fusion (PRD-DM) were treated with either PDGF or with both PDGF and LY were immunoprecipitated with anti-WAVE3 antibody and subjected to immunoblotting with anti-phospho-tyrosine PY20. Phosphorylated WAVE3 is detected in the wild-type PRD (PRD-WT) only in the presence of PDGF, but not in the presence of PDGF inhibitor (LY). Cell lysates were also immunoblotted with anti-WAVE3 and anti-GFP antibodies to show expression of the fusion proteins and the presence of equal amounts of these proteins in the cell lysates (input panels). In all cases, the size of proteins verified by western blot are as expected for the protein fragment. β-Actin is a loading control.

WAVE3 has been established as a major driver of the invasion-metastasis cascade in several cancers, including the one originating from the breast, in part through the regulation of the EMT programs and the maintenance of the CSC niche^14,20,22,26–32^. Our published studies have also shown that phosphorylation of WAVE3 at four tyrosine residues (Fig. 1A) can be achieved upon stimulation with PDGF and activation of phosphatidylinositol 3-kinase (PI3K) signaling pathway^15,16^. Furthermore, we have also shown that the PRD domain of WAVE3 is required for the oncogenic activity of WAVE3 by activating the CSC phenotype in TNBC cells^14^. Whether phosphorylation of PRD is also required for the PRD-mediated activation of WAVE3, has not been reported, as did the potential role of PRD-phosphorylation in the activation of the CSC phenotype and the EMT programs. Two of the four tyrosine residues (Y248 and Y337) are located within the PRD domain of WAVE3 (Fig. 1A). Therefore, we sought to investigate how loss of phosphorylation of these two tyrosine residues within the PRD domain affects the oncogenic activity of WAVE3 in BC. As an initial confirmation of our previously published findings^15,16^, we showed that tyrosine phosphorylation of endogenous (full length) WAVE3 can be detected at basal levels in MDA-MB-231 (Fig. 1B) and 4T1 BC cells (Fig. 1C). Upon stimulation with PDGF, phosphorylation levels of WAVE3 increased by at least fivefold (p < 0.05) in both MDA-MB-231 and 4T1 cells (Fig. 1B,C). Next, to investigate the phosphorylation of the PRD domain of WAVE3, we first depleted the endogenous WAVE3 from MDA-MB-231 and 4T1 cells using CRISPR/Cas9 gene editing. We used two different sgRNAs targeting two different exons of both the human and mouse *WAVE3* gene in MDA-MB-231 and 4T1 cells, respectively^14,15^. We generated pools of cell populations of WAVE3-knockout for both MDA-MB-231 (Fig. 1D) and 4T1 (Fig. 1E) cells. A scrambled (SCRAM) sgRNA was used as a negative control. The knockout efficiency was very high in both cell lines since only less than 5% of residual endogenous WAVE3 protein could be detected in the W3-KO cells (Fig. 1D,E). The WAVE3-knockout derivatives were then transduced to stably express either GFP alone (W3-KO), wild-type WAVE3-PRD-GFP fusion (PRD-WT) or a phospho-mutant WAVE3-PRD-GFP fusion (PRD-DM), where both tyrosine residues Y248 and Y337 (Fig. 1A) were mutated to unphosphorylated phenylalanine, Y248F and Y337F. We used western blot analysis to confirm the expression of GFP and the WAVE3-PRD GFP fusion proteins in both MDA-MB-231 (Fig. 1F) and 4T1 (Fig. 1G). Next, protein lysates from these PDGF-treated cells were subjected to immunoprecipitation with anti-WAVE3 antibody, followed by western blot of the resulting immunocomplexes with anti-phosphotyrosine antibody (PY20) to detect phosphorylation levels of WAVE3-PRD. Of note, the polyclonal WAVE3 antibody we used in our study can also recognize the PRD domain, since the antigenic peptide that was originally used to generate the WAVE3 antibody maps to a sequence of the PRD domain that is unique to WAVE3 and is not present in the other WAVE isoforms. The immunoprecipitation results showed that wild-type PRD (PRD-WT) but not phospho-mutant PRD (PRD-DM) can be detected by PY20 antibody in the WAVE3-immunocomplexes, in both MDA-MB-231 (Fig. 1F) and 4T1 (Fig. 1G) cells, indicating that mutation of the two tyrosine residues resulted in complete loss of phosphorylation of the WAVE3 PRD domain. To confirm that PDGF (PI3K signaling) is indeed required for the phosphorylation of the PRD, first, we show that PDGF-mediated phosphorylation of endogenous WAVE3 is completely lost in both MDA-MB-231 (Fig. 2A) and 4T1 (Fig. 2B) cells after treatment with LY294002, an inhibitor of PDGFR and its downstream effector PI3K pathway. Next, W3-KO MDA-MB-231 (Fig. 2C) or 4T1 (Fig. 2D) cells that were transduced to express either GFP or wild-type WAVE3 PRD domain, were treated with LY294002 (LY), after being stimulated with PDGF, and the cell lysates were analyzed by co-immunoprecipitation with anti-WAVE3 antibody. As expected, wild-type PRD (PRD-WT) showed a strong phosphorylation in the presence of PDGF, while treatment with LY294002 inhibited phosphorylation of PRD in both MDA-MB-231 (Fig. 2C) and 4T1 (Fig. 2D). Therefore, we confirm the involvement of both tyrosine residues Y248 and Y337 in the PDGF-mediated phosphorylation of WAVE3PRD domain.

### Phosphorylation of the WAVE3 PRD domain is required for migration and invasion of BC cells in vitro

**Figure 3:**
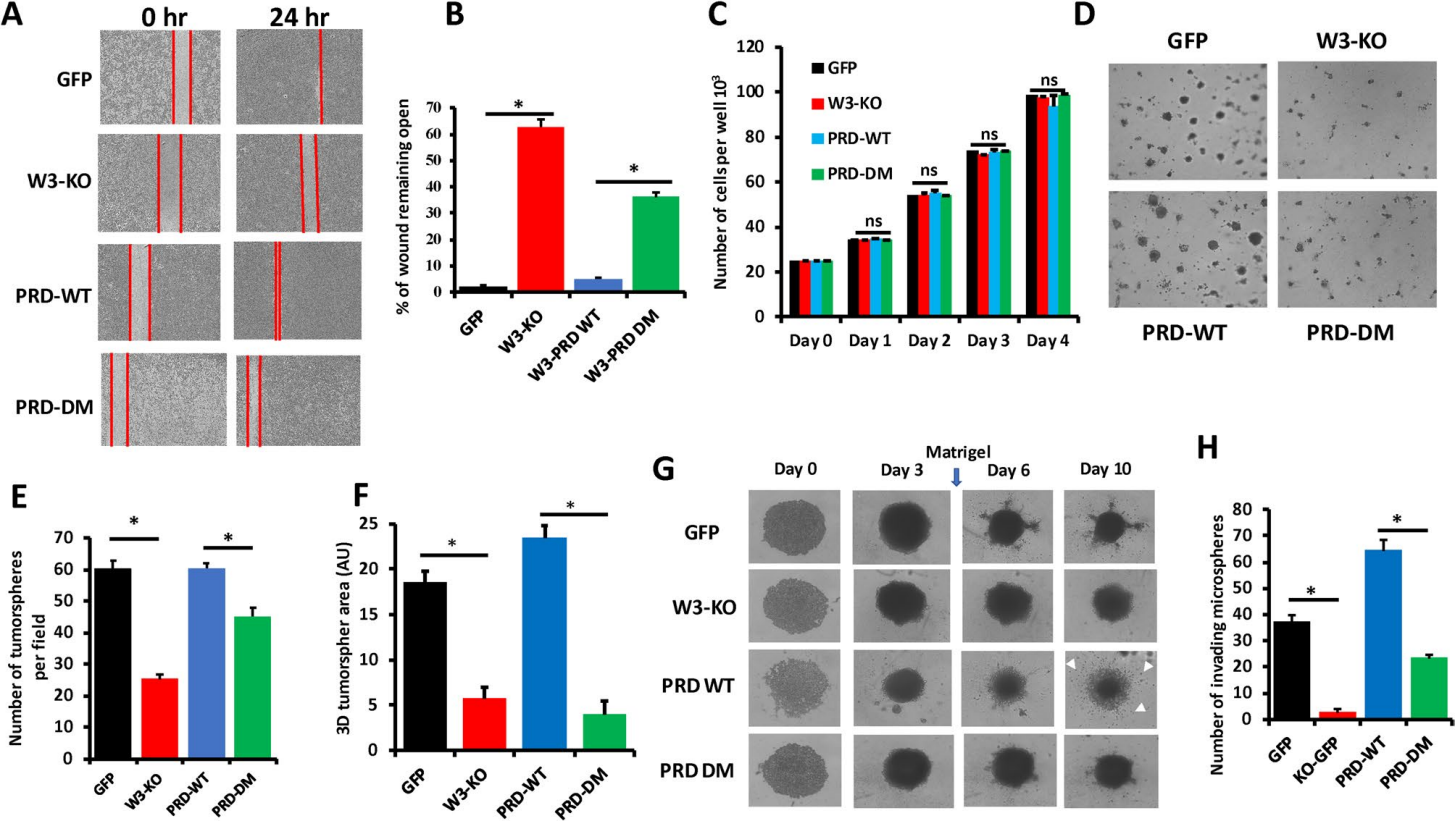
Phosphorylation of the WAVE3 PRD domain is required for migration and invasion of BC cells in vitro. (**A**) Representative micrographs of wound healing assays of confluent cell cultures of parental MDA-MB-231 cells (GFP), their WAVE3-deficient derivatives (W3-KO) or the W3-KO expressing either PRD domain wild-type (PRD-WT) or phospho-mutant (PRD-DM) WAVE3 that were induced to migrate into scratch wounds in confluent monolayers over 24 h. (**B**) Quantification of the unclosed wound (open area) at 24 h from 12 different wounds was measured and plotted as the percentage of the wound at time zero for MDA-MB-231 cells. (**C**) Cell proliferation over 5 days (**D**) Representative micrographs of parental MDA-MB-231 and its derivatives induced to form tumorspheres. (**E**) Quantification of the number of tumorspheres formed per area of the field. (**F**) Quantification of the total area occupied by resulting tumorspheres. (**G**) Representative micrographs of parental MDA-MB-231 and its derivatives induced to form tumorspheres. Single spheres were made in a 96-well ULA plates and Matrigel (2.5 v/v) was added to the tumorsphere cultures at day 3 and images were captured using Incucyte for 10 days. White arrows represent invading microspheres. (H) Quantification of the number of invading microspheres at day 10. Data are the means ± SD (n = 3, * and **, p < 0.05; Student's t-test).

Cancer cell migration and invasion are hallmarks of cancer^33,34^ and the role of WAVE3 in regulating these cellular activities is well documented (Reviewed in^3,5,35^). Our recently published study has shown that WAVE3 phosphorylation is required for the activation of BC cell migration and tumorsphere growth and invasion^15^. Whether the PRD domain of WAVE3 and its phosphorylation are also important in such oncogenic activities have not been reported. Parental MDA-MB-231 or 4T1 (GFP), their W3-KO derivatives expressing either GFP, wild-type PRD or phospho-mutant PRD were subjected to 2-dimensional (2D) wound-healing assay and the extent of the wound that remained open at the end of the experiment was compared between the experimental groups. In the parental (GFP) MDA-MB-231 cells less than 5% of the wound remained open, while in the W3-KO cells more than 70% of wound remained open (Fig. 3A,B). In the WAVE3-deficient cells expressing wild-type PRD (W3-PRD-WT), the wound was almost completely closed, with less than 5% of the wound remained open. However, re-expression of phospho-mutant PRD (W3-PRD-DM) achieved less than 60% of wound closure (Fig. 3A,B). Differences in wound closure were the result of differences in cell migration rather than differences in cell proliferation, since loss of WAVE3 expression, or re-expression of wild-type PRD or phospho-mutant PRD did not affect cell proliferation of MDA-MB-231 (Fig. 3C). Thus, we confirmed that not only expression of the WAVE3-PRD domain, but its phosphorylation is required for cell migration of BC cells. We also used 3-dimensional (3D) tumorsphere growth and invasion assays to assess the role of phosphorylated WAVE3-PRD in tumorsphere growth and invasion in 3D conditions. We found loss of WAVE3 (W3-KO) in MDA-MB-231 cells to significantly (p < 0.05) inhibit the number of tumorspheres (Fig. 3D,E) and their size (Fig. 3F), compared to the parental cells (GFP). However, while re-expression of wild-type PRD (PRD-WT) in the W3-KO cells was able to restore the growth of the tumorspheres to levels comparable to those obtained with the parental cells, re-expression of phospho-mutant PRD (W3-PRD-DM) resulted in tumorsphere growth that was closely similar to that obtained with the W3-KO cells (Fig. 3D–F). We also found loss of WAVE3 to significantly inhibit the MDA-MB-231 invasion of the Matrigel matrix (Fig. 3G,H). Re-expression of wild-type PRD (W3-WT-PRD), but not that of phospho-mutant PRD (W3-PRD-DM) restored invasion into the Matrigel matrix (Fig. 3G,H). Therefore, our findings show that the PRD domain by itself was sufficient to mimic the activity of the full length WAVE3, and that tyrosine phosphorylation of WAVE3-PRD domain if required for the in vitro oncogenic activities of WAVE3 in BC cells in vitro.

### Phosphorylation of the WAVE3 PRD domain is required for the growth of BC primary tumors in vivo

**Figure 4:**
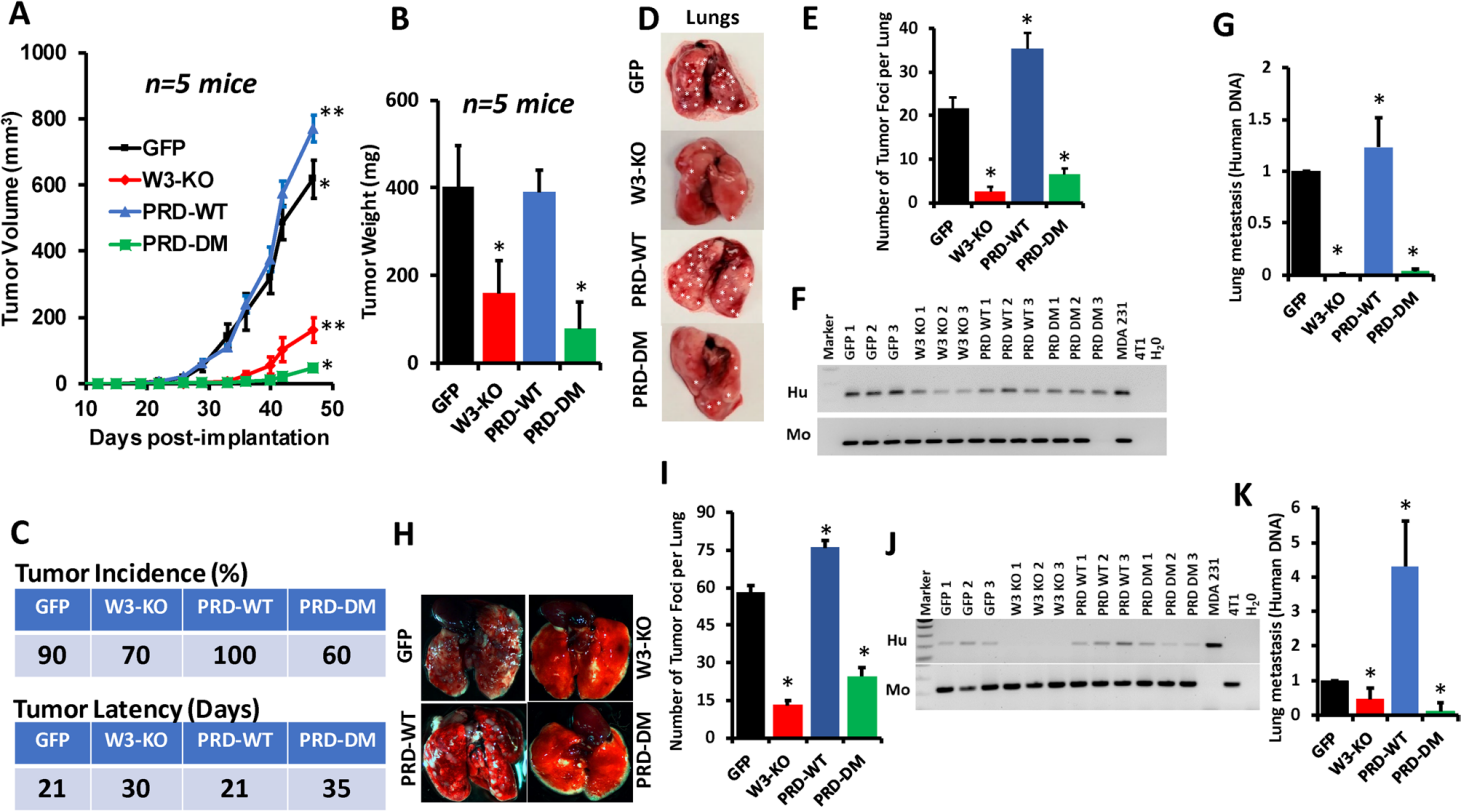
Phosphorylation of the WAVE3 PRD domain is required for growth of primary tumor of BC cells in vivo. (**A**) Quantification of volume of tumors derived from implantation of parental MDA-MB-231 (GFP), WAVE3-deficient (W3-KO) MDA-MB-231 cells, or W3-KO MDA-MB-231 cells that were transfected with wild-type WAVE3-PRD-GFP fusion (PRD-WT) or phospho-mutant WAVE3-PRD-GFP fusion (PRD-DM) into the mammary fat pads of NSG mice. (**B**) Quantification of tumor weights. (**C**) Tumor incidence in percentage and tumor latency in days for each group. (**D**) Representative lungs of NSG mice described in A. Asterisks denote metastasis foci. (**E**) Quantification of lung metastasis foci. (**F**) Analysis of human and mouse genomic PCR followed by agarose gel electrophoresis of DNAs of the lungs from mice described in A. The human-specific ERVK6 gene can be identified in human DNA (MDA-MB-231) but not in mouse DNA (4T1). The presence of the human ERVK6 sequences in the mouse lung tissues indicates the presence of human cells in these tissues and is representative of metastasis. (**G**) Quantification of lung metastasis using qt-Genomic PCR (H) Representative lungs of NSG mice that were injected via tail vein with either parental (GFP) MDA-MB-231, WAVE3-deficient (W3-KO) MDA-MB-231 cells, or W3-KO MDA-MB-231 that were transfected with wild-type WAVE3-PRD-GFP fusion (PRD-WT) or phospho-mutant WAVE3-PRD-GFP fusion (PRD-DM). (**I**) Quantification of lung metastasis foci. (**J**) Analysis of human and mouse genomic PCR followed by agarose gel electrophoresis of DNAs of the lungs from mice described in G. (**K**) Quantification of lung metastasis using qt-Genomic PCR.

To further assess the effects of loss of phosphorylation in the WAVE3-PRD domain on tumor growth and metastasis in vivo, mammary fat pads of NOD-scid-IL2Rgamma knockout (NSG) mice were inoculated with parental MDA-MB-231 cells (GFP), W3-KO, or W3-KO re-expressing either wild-type PRD domain (W3-WT-PRD) or phospho-mutant PRD domain (W3-PRD-DM), and tumor growth was assessed over 8 weeks. Loss of WAVE3 inhibited the growth of primary tumors (Fig. 4A,B). Re-expression PRD-WT, not only restored tumor growth, but enhanced tumor growth to levels that were significantly higher (p < 0.05) than those obtained with the parental group, while re-expression of PRD-DM resulted in tumor growth comparable to that obtained with the W3-KO group (Fig. 4A,B). Not every mouse in every group developed tumors (tumor incidence) ∼ 8 weeks post tumor cells implantation (Fig. 4C). While 90% and 100% of mice implanted with parental MDA-MB-231 and PRD-WT cells, respectively, developed tumors, only 70% and 60% of mice implanted with W3-deficient and PRD-DM cells, respectively, developed tumors (Fig. 4C). The reason for not achieving 100% tumor incidence in the control group may simply be linked to a technical failure during the injection process, since that same mouse was injected on both the right and left abdominal mammary glandes; the left side developed a large tumor, while the right side did not. Tumor latency was also affected as a result of loss of WAVE3 or re-expression of PRD-DM; tumors in these mice were palpable 30 and 35 days post implantation, while in the mice implanted with the parental cells or the PRD-WT cells tumor latency was reduced to 21 days post implantation (Fig. 4C). Tumor burden, as assessed by tumor volume (Fig. 4A) and weight (Fig. 4B), was significantly lower (p < 0.05) in the mice implanted with the WAVE3-deficient cells and the WAVE3-deficient re-expressing phospho-mutant PRD. Thus, loss of phosphorylation the WAVE3 PRD domain inhibits the rate of primary tumor growth in vivo. These differences in tumor burden were not a result of changes in tumor cell proliferation by manipulation of WAVE3 expression of post-translational modification, as there was no significant difference in the number of viable cells between the groups of cells in the course of 5 days (Fig. 3C).

### Phosphorylation of the WAVE3 PRD domain is required for BC metastasis in both the spontaneous and the experimental metastasis assays

Next, we sought to investigate how the loss of phosphorylation of the WAVE3-PRD domain influences BC metastasis. In the spontaneous metastasis assay, after the primary tumors were excised from the mammary gland sites, the mice were sacrificed, lungs removed, and lung metastasis nodules counted. We found the number of lung nodules (metastasis foci), was reduced by more than tenfold (p < 0.01) in mice implanted with the WAVE3-deficient (W3-KO) cells compared to their parental (GFP) counterparts (Fig. 4D–G). In the lungs of mice implanted by the W3-KO cells re-expressing the wild-type PRD (W3-PRD-WT), the number of lung metastasis foci was similar to that of the parental (GFP) group, while in the lungs of mice implanted by the W3-KO cells re-expressing phospho-mutant PRD (W3-PRD-DM), the number of lung metastasis foci was close to that seen in the W3-KO group (Fig. 4D–G).

We also used the lung colonization (experimental metastasis) assay, in which cancer cells were injected directly in the blood via the lateral tail vein to determine the effect of loss of phosphorylation of the WAVE3-PRD domain on metastasis. Here again (Fig. 4H–K) we found the lung metastasis nodules were reduced by ~ ten-fold (p < 0.01) when WAVE3-deficient (W3-KO) MDA-MB-231 or the W3-KO cells re-expressing the phospho-mutant WAVE3-PRD (W3-PRD-DM) were injected into mice, compared to parental (GFP) or the W3-KO cells re-expressing wild-type PRD (W3-PRD-WT). These results show that phosphorylation of the WAVE3-PRD domain plays a critical role in the invasion and metastasis of BC tumors as demonstrated here in both the spontaneous and experimental metastasis assays.

### Phosphorylation of the WAVE3 PRD domain is required for the interaction between WAVE3 and YB1

**Figure 5:**
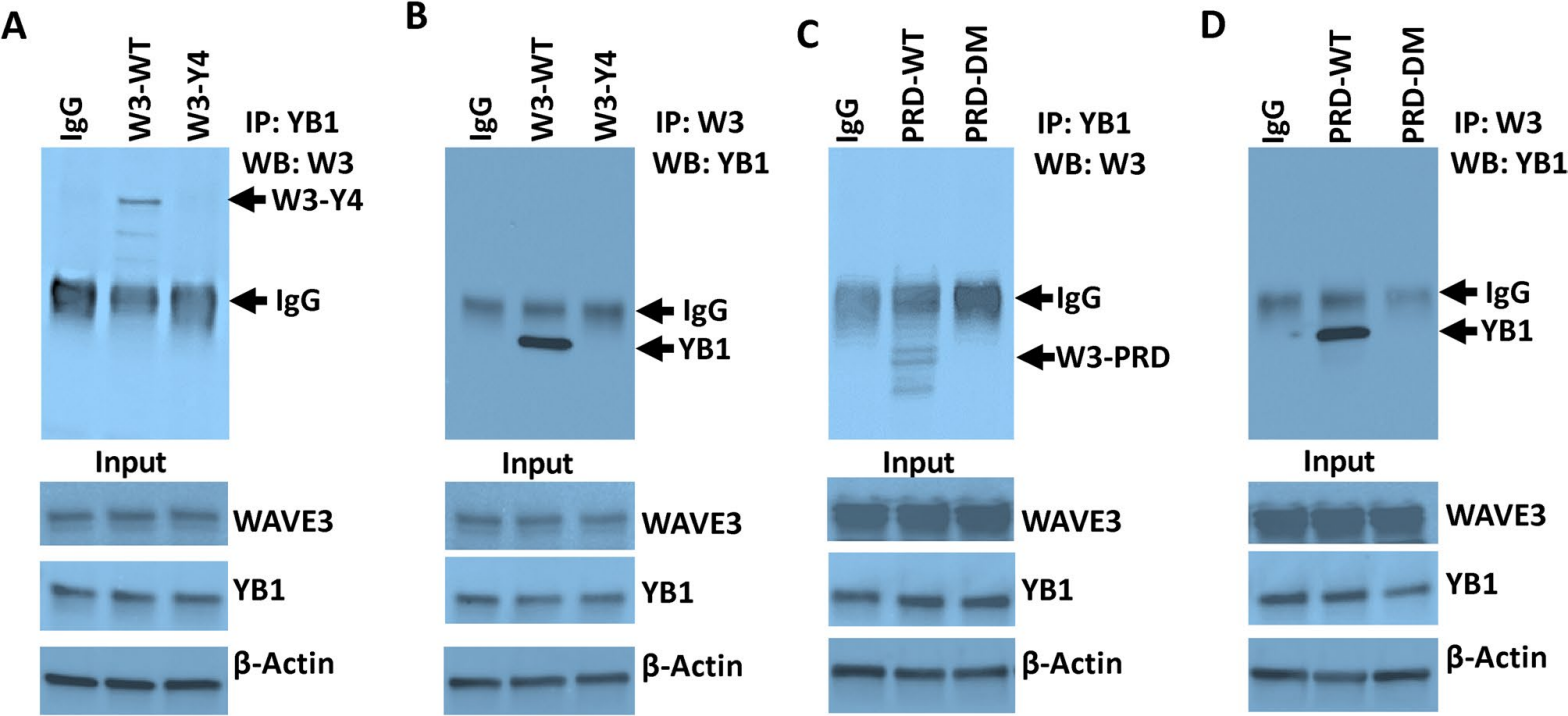
Phosphorylation of the WAVE3 PRD domain is required for the interaction between WAVE3 and YB1. (**A**,**B**) Protein lysates from WAVE3-deficient MDA-MB-231 cells expressing either wild-type WAVE3 (W3-WT) or phospho-mutant WAVE3 where all 4 tyrosine residues were mutated to phenylalanine (W3-Y4) were immunoprecipitated with (**A**) anti-YB1 antibody and subjected to immunoblotting with anti-WAVE3 or (**B**) anti-WAVE3 antibody and subjected to immunoblotting with anti-YB1 to detect phosphorylated W3-Y4 or YB1 (C and D) Protein lysates from WAVE3-deficient MDA-MB-231 cells expressing either wild-type PRD (PRD-WT) or phospho-mutant PRD (PRD-DM) were immunoprecipitated with (**C**) anti-YB1 antibody and subjected to immunoblotting with anti-WAVE3 or (**D**) anti-WAVE3 antibody and subjected to immunoblotting with anti-YB1 to detect phosphorylated W3-PRD or YB1. Cell lysates were sequentially immunoblotted with either anti-WAVE3 or anti-YB1 antibodies to show the presence of equal amounts of the corresponding proteins in the cell lysates.

YB1 is a transcription factor that was shown to regulate the transcription of CSCs-specific genes such as Oct4, Nanog and Sox2^14,36,37^. Our published studies showed that the interaction between WAVE3 and YB1 is required for the translocation of YB1 to the nucleus to allow YB1 to exert its transcription factor function^14^. We have also shown that interaction between YB1 and the PRD of WAVE3 is essential for the nuclear translocation of YB1 and for the regulation of transcription of the CSCs-specific genes^14^. Here, we sought to investigate whether phosphorylation of WAVE3 and its PRD domain are required for the interaction with YB1. First, we used co-immunoprecipitation of cell lysates from WAVE3-deficient MDA-MB-231 cells that stably express either wild-type WAVE3 (W3-WT) or phospho-mutant WAVE3 (W3-Y4F) to show that when all four tyrosine residues are mutated to unphosphorylated phenylalanine (W3-Y4F)^15,16^, the interaction between WAVE3 and YB1 is completely lost (Fig. 5A,B). Thus, we show that WAVE3 phosphorylation is required for the WAVE3-YB1 interaction. Next, we established that wild-type PRD is required for the interaction between WAVE3 and YB1 since loss of phosphorylation of the PRD domain (W3-PRD-DM) inhibited such interaction (Fig. 5C,D). Therefore, our data confirm that phosphorylation of the WAVE3-PRD domain is required for the WAVE3-YB1 interaction.

### Phosphorylation of the WAVE3 PRD domain is required for the WAVE3-mediated regulation of the EMT programs and for the YB1-mediated regulation of the cancer stem cell niche

**Figure 6:**
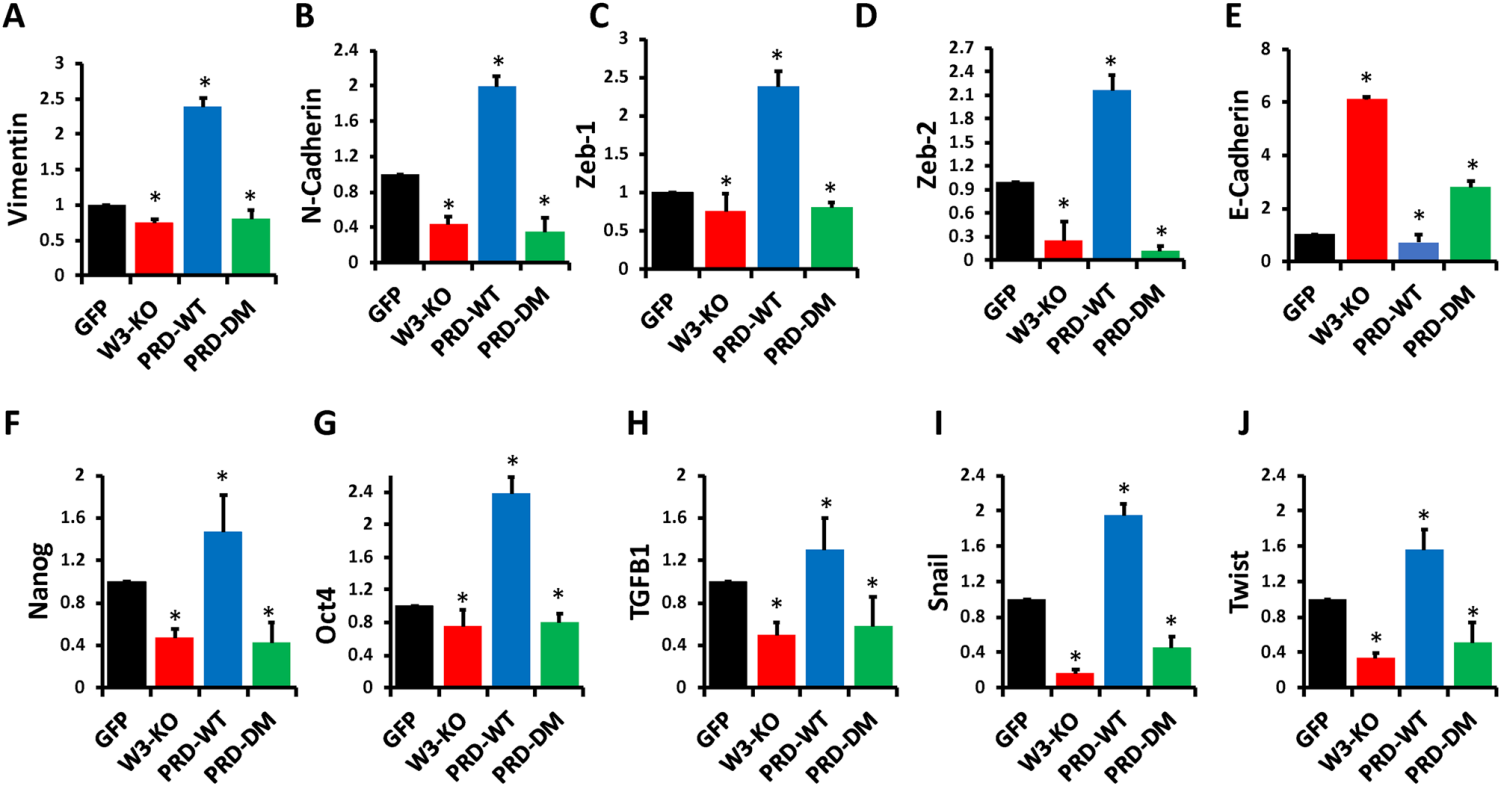
Phosphorylation of the WAVE3 PRD domain is required for the WAVE3-mediated regulation of the EMT-program and for the YB1-mediated regulation of the cancer stem cell niche: qt-RT-PCR data. Quantitative RT-PCR of mRNA of (**A**) Vimentin (**B**) N-Cadherin (**C**) Zeb-1 (**D**) Zeb-2 (**E**) E-Cadherin (**F**) Nanog (**G**) Oct4 (**H**) TGFβ1 (**I**) Snail and (**J**) Twist from parental (GFP) MDA-MB-231, WAVE3-deficient (W3-KO) MDA-MB-231 cells, or W3-KO MDA-MB-231 cells that were transfected with either wild-type WAVE3-PRD-GFP fusion (PRD-WT) or phospho-mutant WAVE3-PRD-GFP fusion (PRD-DM). GAPDH was used for normalization. Data are the means ± SD, N = 3, * represents p < 0.05 compared to GFP, Student’s t-test.

We further investigated the biological significance of phosphorylation of the WAVE3-PRD domain which relate the oncogenic activity of WAVE3 and YB1. Our previous studies showed that WAVE3 is involved in the activation of the EMT programs^28,32^. Here we show that loss of WAVE3 in MDA-MB-231 (W3-KO) results in a significant (p < 0.05) decrease in expression levels of EMT markers Vimentin (Fig. 6A), N-cadherin (Fig. 6B), Zeb-1 (Fig. 6C) and Zeb-2 (Fig. 6D), while expression levels of the mesenchymal-to-epithelial (MET) marker E-Cadherin were increased ~ six-fold (Fig. 6E), compared to the parental (GFP) cells. Re-expression of the wild-type WAVE3-PRD domain (PRD-WT), not only restored expression of these markers to levels seen in the parental cells (GFP), but expression levels of Vimentin, N-cadherin, Zeb-1 and Zeb-2 were increased by 2 to 2.5-fold (p < 0.05) compared the parental cells (Fig. 6A–D, respectively). Conversely, overexpression of wild-type PRD resulted in ~ two-fold decrease (p < 0.05) in expression levels of E-Cadherin compared to the parental cells (Fig. 6E). On the other hand, re-expression of phospho-mutant PRD (PRD-DM) failed to restore expression of Vimentin, N-Cadherin, Zeb-1 and Zeb-2 to the levels seen in the parental cells. In fact, expression levels of these markers in the phospho-mutant group were close to the ones observed in the WAVE3-deficient (W3-KO) cells than to those observed in the parental cells.

**Figure 7:**
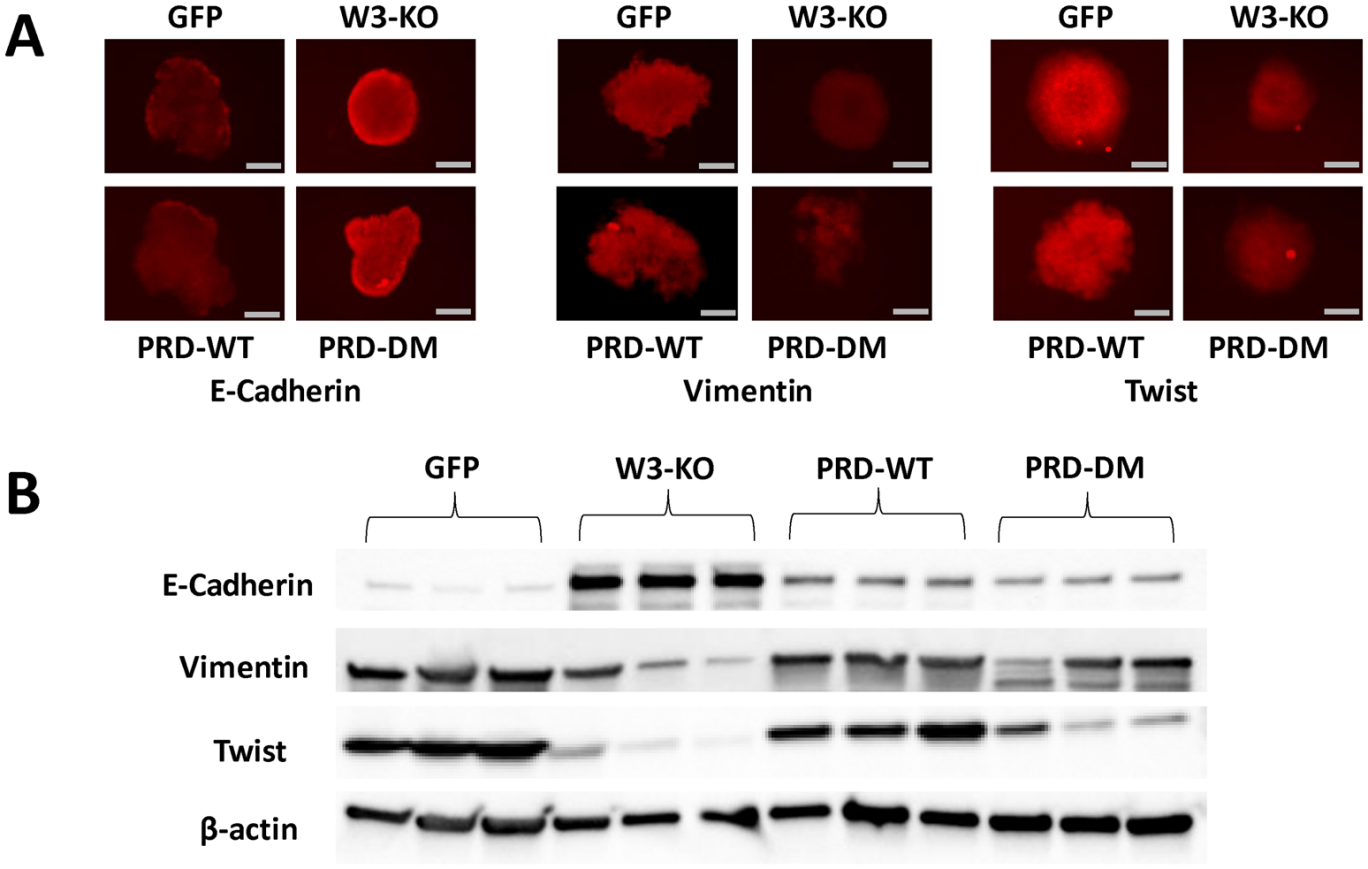
Phosphorylation of the WAVE3 PRD domain is required for the WAVE3-mediated regulation of the EMT-program and for the YB1-mediated regulation of the cancer stem cell niche: Immunofluorescence and Western Blot data. (**A**) Representative micrographs of tumorspheres derived from parental 4T1 and its derivatives and immunostained with antibodies against the indicated proteins. Scale bar: 150 µm. (**B**) Western blots developed with the indicated antibodies of protein lysates from the primary tumors of mice implanted with the indicated MDA-MB-231 cells and their derivatives. β-Actin was used a loading control.

Similar trends were observed with the CSC markers. Our published work established that both WAVE3 in YB1 are required for maintenance of the CSC and that the PRD domain of WAVE3 was critical for the activation of expression of CSCs^14^. Here we found that not only the expression of the WAVE3 PRD domain, but its phosphorylation is essential for the expression of CSC markers. Loss of WAVE3 (W3-KO) in MDA-MB-231 cells resulted in a significant (p < 0.05) inhibition of Nanog (Fig. 6F) and Oct4 (Fig. 6G), two well established CSC markers the expression of which are driven by YB1 transcription factor. Expression of other markers that are known to be associated with the CSC phenotype, such as TGFβ1 (Fig. 6H), Snail (Fig. 6I) and Twist (Fig. 6J) was also inhibited as a result of loss of expression of WAVE3. Re-expression of wild-type PRD (PRD-WT) increased expression of these markers to levels that were even higher than those observed in the parental cells, while re-expression of phospho-mutant PRD (PRD-DM) failed to restore expression of these CSC markers to levels observed in the parental cells. These results were validated using immunofluorescence assays (Fig. 7 and Sup Fig. S1-S3) of tumorspheres derived from parental 4T1 (GFP), their WAVE3-deficient (W3-KO), W3-KO 4T1 re-expressing wither wild-type PRD (PRD-WT) or phosphomutant PRD (PRD-DM). Loss of E-Cadherin is a hallmark of EMT, while it’s gain is a hallmark of MET. Consistent with the role of WAVE3 in the regulation of the EMT programs^28,38^, we found a significant increase of E-Cadherin staining in the W3-KO-derived tumorspheres, compared to those derived from the parental cells (Fig. 7A, left panel and Sup Fig. S1). Overexpression of phosphomutant PRD mimicked the effect of loss of WAVE3 expression, while overexpression of PRD-WT did not have any noticeable effect on E-Cadherin staining. The Opposite scenario was observed with Vimentin, the expression of which is a marker of EMT; Vimentin staining was noticeably higher in the parental cells and the PRD-WT-expressing cells (Fig. 7A, middle panel and Sup Fig. S2). Finally, we found Twist staining to be lost as a result of loss of WAVE3 expression or expression of PRD-DM (Fig. 7A, right panel and Sup Fig. S3). We further confirmed these results by Western Blot of protein lysates of tumors derived from mice injected with parental (GFP), WAVE3-deficient (W3-KO), W3-KO re-expressing wild-type PRD (PRD-WT) or phospho-mutant PRD (PRD-DM) MDA-MB-231 cells (Fig. 7B). Together, these data clearly established that phosphorylation of the WAVE3-PRD domain is essential for regulation of the EMT programs as well as the maintenance of the CSC phenotype.

## Discussion

This study demonstrates that loss of tyrosine phosphorylation of the WAVE3 PRD domain is sufficient to inhibit several oncogenic activities of WAVE3 in TNBC cells. Loss of tyrosine phosphorylation of full length WAVE3 was found to inhibit the oncogenic activity of WAVE3 in TNBC^15^. The present study identifies a previously undocumented function of the PRD domain of WAVE3 that depends on its phosphorylation at two specific tyrosine residues. As a consequence of this post-translational modification, several hallmarks of cancer were affected, including sustained proliferative signaling, resisting cell death, activation of the invasion-metastasis cascade and evading growth suppressors^33,34^. In fact, we showed that inhibition of phosphorylation of WAVE3 PRD mimicked the loss of expression of WAVE3 by severely inhibiting cell migration, invasion and 3D tumorsphere growth and invasion, in vitro as well tumor growth, invasion and metastasis in vivo. All of these cancer phenotypes have been shown to be affected by either loss of expression of WAVE3 or its phosphorylation^14–18,20–22,26,29–32,39–41^. We applied a combination of molecular, genetic and pharmacologic manipulations, as well as different biochemical and cell imaging analyses in vitro, in addition to in vivo mouse models for TNBC, to investigate the role of phosphorylation of the WAVE3 PRD domain in the WAVE3-mediated regulation of cancer growth and metastasis in BC.

In this study, we also demonstrated that phosphorylation of WAVE3 PRD domain is required for the activation of the CSC phenotype and the EMT programs. Our previous studies have reported that WAVE3 phosphorylation is required for the activation of cell migration, tumorsphere growth and invasion in TNBC^15^. However, according to our knowledge, this study provides the first evidence that not only phosphorylation of WAVE3, but phosphorylation of PRD domain is also required for the oncogenic activities of WAVE3. In addition, we showed that PDGF (PI3K signaling) is required for the phosphorylation of WAVE3 PRD domain. We presented evidence that phosphorylation of WAVE3 at both tyrosine residues Y248 and Y337 can be attained by PDGF downstream of PI3K. In fact, the activation of the PI3K by PDGF is not direct, but instead is mediated downstream of PDGFR^42^. Inhibition of WAVE3 phosphorylation by LY294002 treatment is in fact mediated through the inhibition of PDGFR, which in turn results in inhibition PI3K and its downstream effectors, such as the c-Abl non-receptor tyrosine kinase, which we showed is directly involved in WAVE3 phosphorylation^16^. Several studies have shown that, in addition to PI3K/c-Abl, WAVE3 phosphorylation can also be achieved downstream of TGF-β and EGF^15^ as well as JAK1/2^17^. WASP, a WAVE3-related protein was also shown to be phosphorylated by members of the Src family kinases^43^, while N-WASP can be activated downstream of tyrosine kinases in a PIP2-depedent manner^44^. On the other hand, WAVE1 (WASF1) can be activated through its phosphorylation by MAP kinases^45^. Whether WAVE3 can be phosphorylated by this family of kinases is however not known. We are, however, very confident that the signaling axis of PI3K is a major activator of WAVE3 phosphorylation, since treatment of PDGF-stimulated MDA-MB-231 cells with the PI3Kα-specific inhibitor alpelisib^46^, almost completely abrogated WAVE3 tyrosine phosphorylation^15^.

Using 2D wound-healing assay and 3D tumorsphere growth and invasion assays, we also showed that phosphorylation of the WAVE3 PRD domain enhances tumor growth and metastasis, while loss of phosphorylation in the phospho-mutant WAVE3 PRD domain inhibited these oncogenic properties. WAVE3 consists mainly of three distinct subdomains: the N-terminus Basic Region (BR), a central proline-rich domain (PRD) and the distal Verprolin Cofilin Acidic (VCA) domain^4,47^ and (Fig. 1A). The VCA domain, which is conserved in all 5 members of the WASP/WAVE3 family of proteins^47^, is responsible for the binding to the actin-related protein (Arp1/2) complex to activate elongation of actin filaments^4,47^. The BR, which is ~ 200 amino acid long is loosely conserved between the three members of the WAVE family, and its specific function is still largely unknown, expect for its speculated auto-inhibitory effect on the WAVE regulatory complex (WRC), where it was postulated that folding of this BR domain masks the binding of RAC-GTP to WAVE3 that is required for the activation of the WRC^9,48^. The PRD domain is also not very well conserved among the WAVE family^4,47,49^. More importantly, the two tyrosine residues Y248 and Y337 are unique to the PRD domain of WAVE3 and are conserved in both the human and the mouse WAVE3 proteins, but not found in the other WASP/WASF family members^47^, which makes the function of this domain specific to WAVE3. The Y-box-binding protein-1 (YB1) has a dual function. In the cytoplasm, YB1 acts as a regulator mRNA translation and stability, while in the nucleus YB1 functions as a transcription factor that regulates expression of several CSC-specific genes^50–52^. Our previous studies showed that interaction between YB1 and WAVE3 is required for the translocation of YB1 from cytoplasm to the nucleus, since YB1 does not have a nuclear translocation signal. In this instance WAVE3 plays the role of a shuttle protein that transports YB1 inside the nucleus to allow it to perform its nuclear transcription function and activation of expression of the CSC-specific transcription factors, Oct4 and Nanog and Sox^14^. Accordingly, we found WAVE3 to play a critical role in the maintenance of CSC niche through its interaction with YB1^14^. Here, we showed that expression of the CSC-specific transcription factors, Oct4 and Nanog, is significantly diminished as a result of loss of expression of WAVE3 or phosphorylation of its PRD domain, therefore, demonstrating the requirement of PRD and its phosphorylation in the regulation of CSC maintenance. Finally, it has now been widely demonstrated that the oncogenic activity of WAVE3 is closely associated with numerous hallmarks of cancer, including EMT (Reviewed in^3,5^). Our results found that loss of WAVE3 expression or loss of phosphorylation of its PRD domain inhibit the EMT programs through downregulation of vimentin, N-cadherin, ZEB1 and ZEB2, and upregulation of E-cadherin. In addition, the miR200 family are known master regulators of EMT^53–57^. Our previous studies have shown that the WAVE3-mediated modulation of the EMT programs is regulated downstream of the miR200 family^28^. While the studies by Burk et al.^53^ and Korpal et al.^57^ have identified a negative feed-back loop between ZEB1/ZEB2 and members of the miR200 family that regulates EMT and cancer cell invasion, while the report by Teng and colleagues have shown that WASF3 (WAVE3) regulates the ZEB1-mediated inhibition of miR-200, and the subsequent activation of BC invasion cells^29^. The involvement of the phosphorylated WAVE3 and its PRD domain in the regulation of ZEB1 and ZEB2 further cements the major role that WAVE3 plays in the very complex biological process such as EMT. Together, our findings show that phosphorylation of WAVE3 PRD domain is important for invasion and metastasis of TNBC tumors, and is required maintenance of CSC population. Therefore, targeted inhibition of phosphorylation of the PRD domain of WAVE3 may represent a novel therapeutic option for the inhibition of TNBC progression and metastasis.

## Supplementary Information

Supplementary Information 1.

Supplementary Information 2.
