# Publisher Correction: Phosphorylation of the proline-rich domain of WAVE3 drives its oncogenic activity in breast cancer

**DOI:** 10.1038/s41598-021-94981-0

**Published:** 2021-07-29

**Authors:** Urna Kansakar, Wei Wang, Vesna Markovic, Khalid Sossey‑Alaoui

**Affiliations:** 1 Department of Medicine, Rammelkamp Center for Research, MetroHealth, Cleveland, OH USA; 2 Case Western Reserve University School of Medicine, Cleveland, OH USA; 3 Case Comprehensive Cancer Center, Cleveland, OH USA; 4 Department of Medicine, Case Western Reserve University School of Medicine, Rammelkamp Center for Research, R457, 2500 MetroHealth Drive, Cleveland, OH 44109 USA

Correction to: *Scientific Reports* 10.1038/s41598-021-83479-4, published online 16 February 2021

The original version of this Article contained an error in Figure 7A, where the image for Twist (GFP) is a duplication of the image for E-Cadherin (W3-KO). The original Figure 7 and accompanying legend appear below.

**Figure 7:**
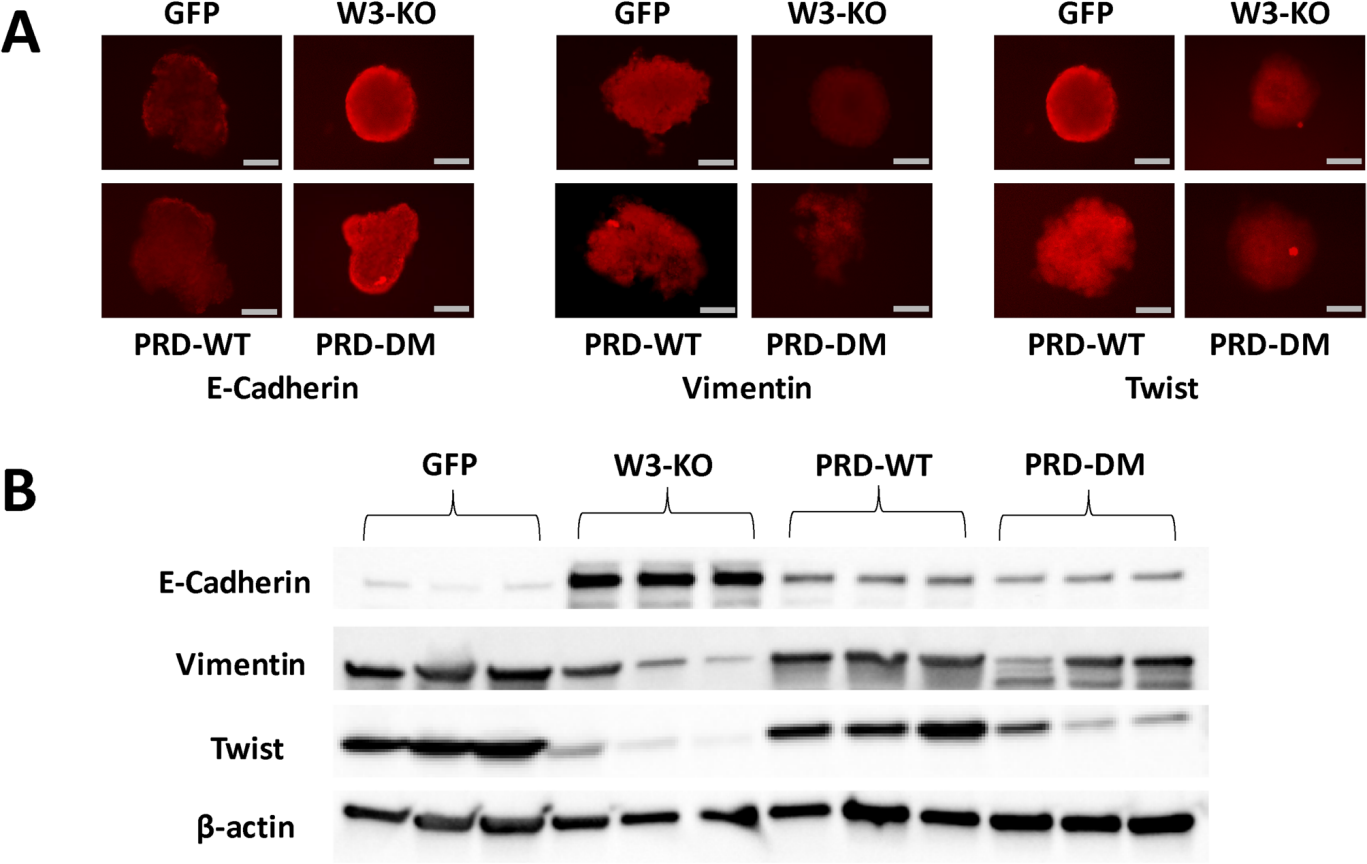
Phosphorylation of the WAVE3 PRD domain is required for the WAVE3-mediated regulation of the EMT-program and for the YB1-mediated regulation of the cancer stem cell niche: Immunofluorescence and Western Blot data. (**A**) Representative micrographs of tumorspheres derived from parental 4T1 and its derivatives and immunostained with antibodies against the indicated proteins. Scale bar: 150 μm. (**B**) Western blots developed with the indicated antibodies of protein lysates from the primary tumors of mice implanted with the indicated MDA-MB-231 cells and their derivatives. β-Actin was used a loading control.

