# Supplementary material for: Phosphorylation of the proline-rich domain of WAVE3 drives its oncogenic activity in breast cancer
Source: Sci Rep. 2021 Feb 16;11:3868. doi: 10.1038/s41598-021-83479-4 (PMC7887190; doi:10.1038/s41598-021-83479-4)
Supplement: Supplementary file 1 — Supplementary Information 1. [file 41598_2021_83479_MOESM1_ESM.pdf]

Phosphorylation of the proline-rich domain of WAVE3 drives its oncogenic activity in breast cancer.

Urna Kansakar[1, 2], Wei Wang[1, 2], Vesna Markovic[1], Khalid Sossey-Alaoui[1, 2, 3,*].

*Corresponding author

[1]Department of Medicine, Rammelkamp Center for Research, MetroHealth, [2]Case Western Reserve University School of Medicine, [3]Case Comprehensive Cancer Center, Cleveland, Ohio, USA.

*Corresponding author:

Khalid Sossey-Alaoui, Ph.D.

Department of Medicine

Case Western Reserve University School of Medicine

Rammelkamp Center for Research, R457

2500 MetroHealth Drive, Cleveland, OH 44109

Phone: 216-778-5275 (office), 216-778-2429 (lab)

FAX: 216-778-4321

email: kxs586@case.edu

email: ksosseyalaoui@metrohealth.org

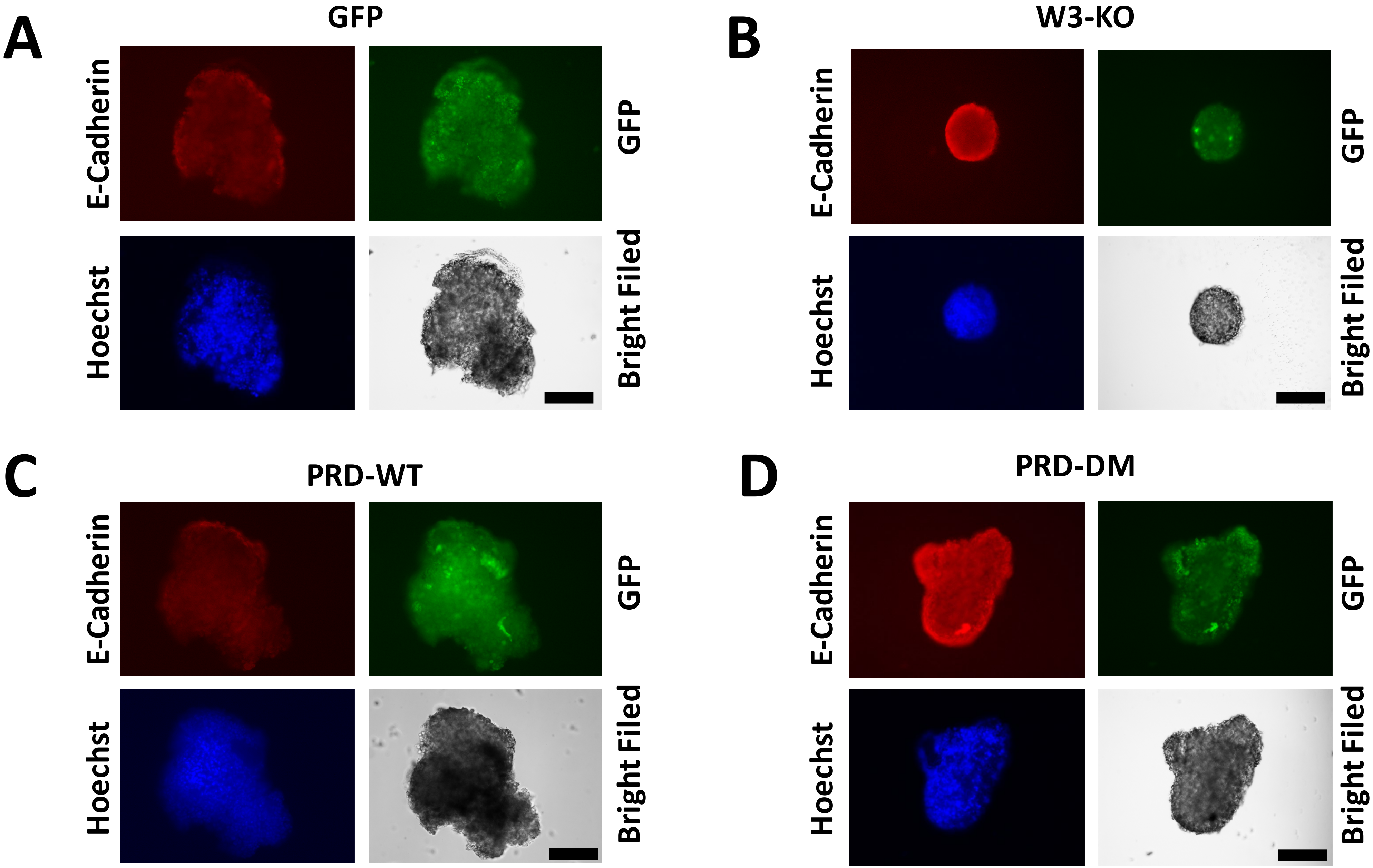
A
GFP
E-Cadherin
GFP
Hoechst
Bright Filed
B
W3-KO
E-Cadherin
GFP
Hoechst
Bright Filed
C
PRD-WT
E-Cadherin
GFP
Hoechst
Bright Filed
D
PRD-DM
E-Cadherin
GFP
Hoechst
Bright Filed

Sup Fig. 1

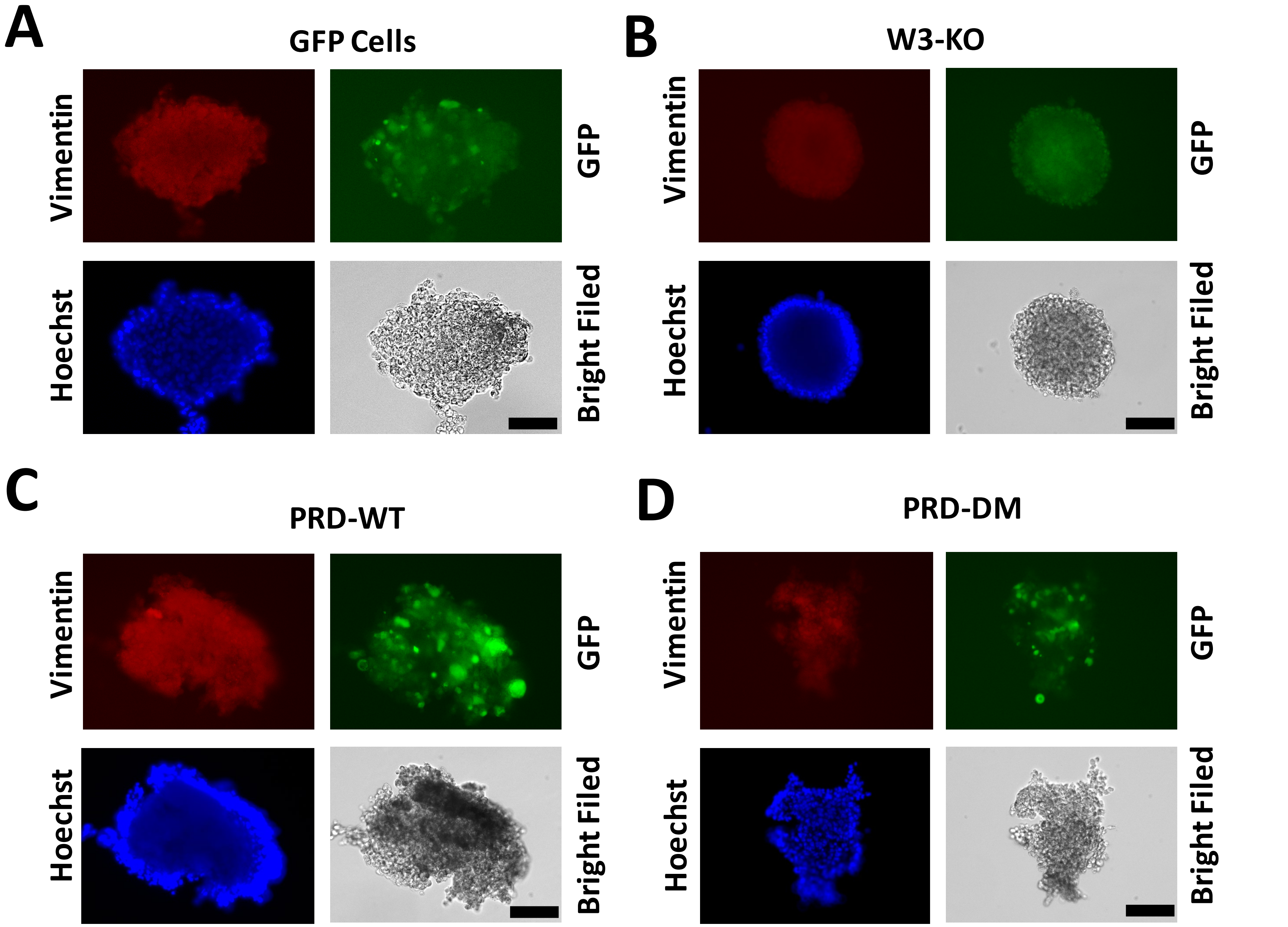
A
GFP Cells
Vimentin
GFP
Hoechst
Bright Filed
B
W3-KO
Vimentin
GFP
Hoechst
Bright Filed
C
PRD-WT
Vimentin
GFP
Hoechst
Bright Filed
D
PRD-DM
Vimentin
GFP
Hoechst
Bright Filed

Sup Fig. 2

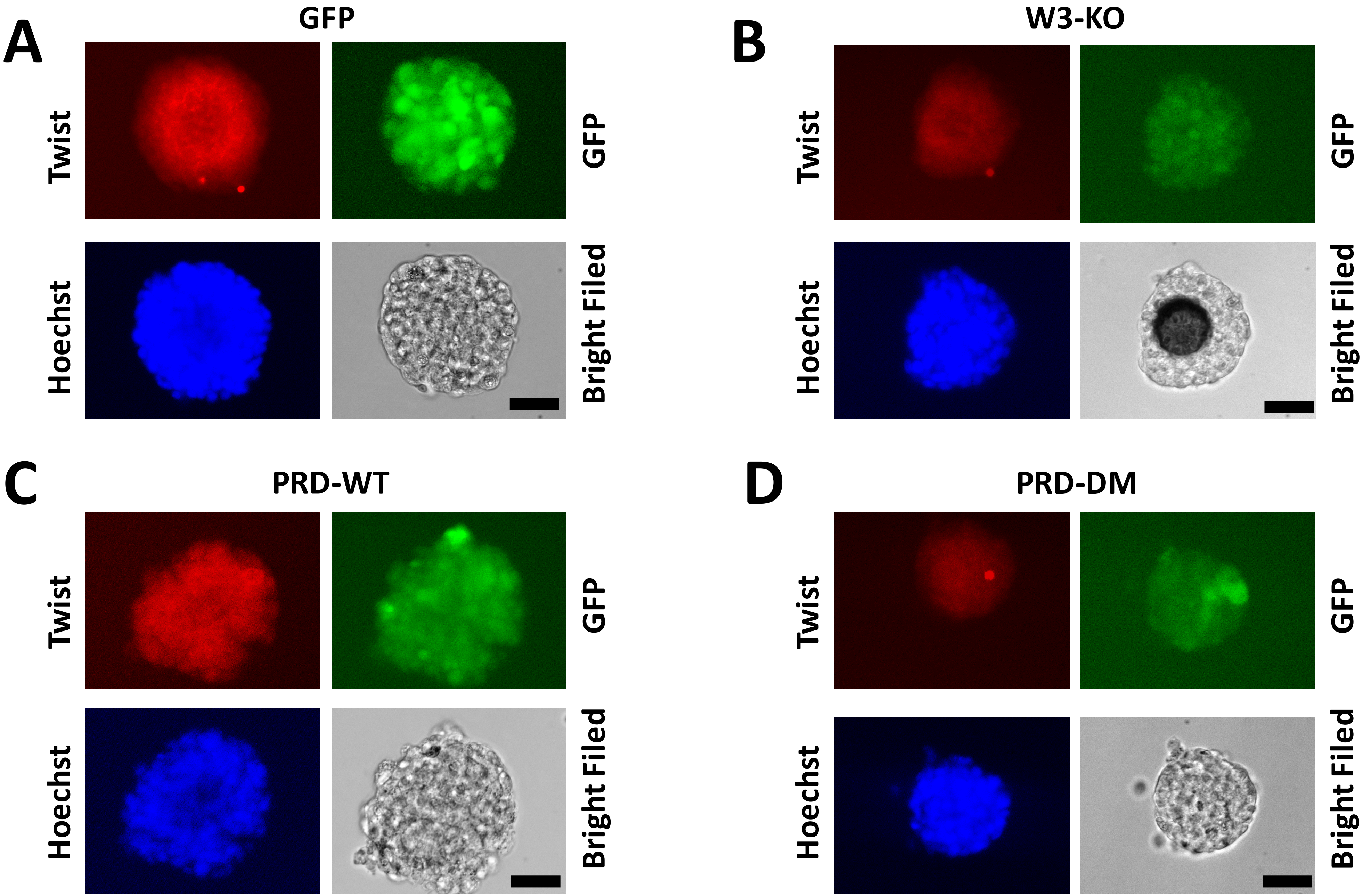
A
GFP
Twist
GFP
Hoechst
Bright Filed
B
W3-KO
Twist
GFP
Hoechst
Bright Filed
C
PRD-WT
Twist
GFP
Hoechst
Bright Filed
D
PRD-DM
Twist
GFP
Hoechst
Bright Filed

Sup Fig. 3

Legends to Supplemental Figures

Supplemental Figure 1

Representative micrographs immunofluorescence staining of tumorspheres derived from (A) parental 4T1 (GFP), W3-deficient (W3-KO) 4T1 cells (B), W3-KO 4T1 cells expressing wild type PRD (PRD-WT) (C), and W3-KO 4T1 cells expressing phosphomutant PRD (PRD-DM) (D). GFP-expressing tumorspheres (green) were immunostained for E-Cadherin (red) and nuclei were counterstained with Hoechst (blue). Bright field snapshots are also shown. Scale bar: 150 µm.

Supplemental Figure 2

Representative micrographs immunofluorescence staining of tumorspheres derived from (A) parental 4T1 (GFP), W3-deficient (W3-KO) 4T1 cells (B), W3-KO 4T1 cells expressing wild type PRD (PRD-WT) (C), and W3-KO 4T1 cells expressing phosphomutant PRD (PRD-DM) (D). GFP-expressing tumorspheres (green) were immunostained for Vimentin (red) and nuclei were counterstained with Hoechst (blue). Bright field snapshots are also shown. Scale bar: 150 µm.

Supplemental Figure 3

Representative micrographs immunofluorescence staining of tumorspheres derived from (A) parental 4T1 (GFP), W3-deficient (W3-KO) 4T1 cells (B), W3-KO 4T1 cells expressing wild type PRD (PRD-WT) (C), and W3-KO 4T1 cells expressing phosphomutant PRD (PRD-DM) (D). GFP-expressing tumorspheres (green) were immunostained for Twist (red) and nuclei were counterstained with Hoechst (blue). Bright field snapshots are also shown. Scale bar: 150 µm.
