# Supplementary material for: Phosphorylation of the proline-rich domain of WAVE3 drives its oncogenic activity in breast cancer
Source: Sci Rep. 2021 Feb 16;11:3868. doi: 10.1038/s41598-021-83479-4 (PMC7887190; doi:10.1038/s41598-021-83479-4)

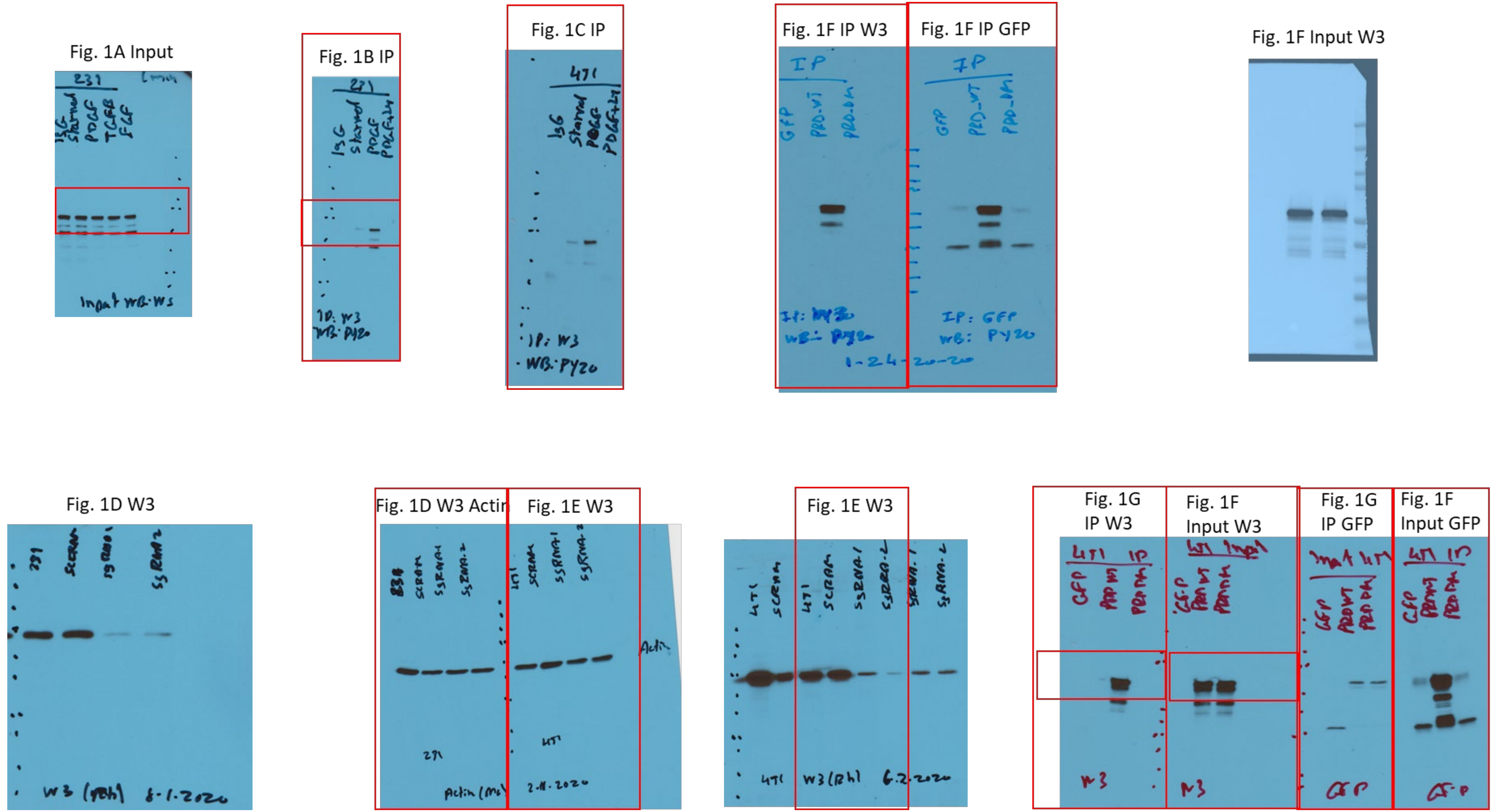
Fig. 1A Input
Fig. 1B IP
Fig. 1C IP
Fig. 1F IP W3
Fig. 1F IP GFP
Fig. 1F Input W3
Fig. 1D W3
Fig. 1D W3 Actin
Fig. 1E W3
Fig. 1E W3
Fig. 1G IP W3
Fig. 1F Input W3
Fig. 1G IP GFP
Fig. 1F Input GFP

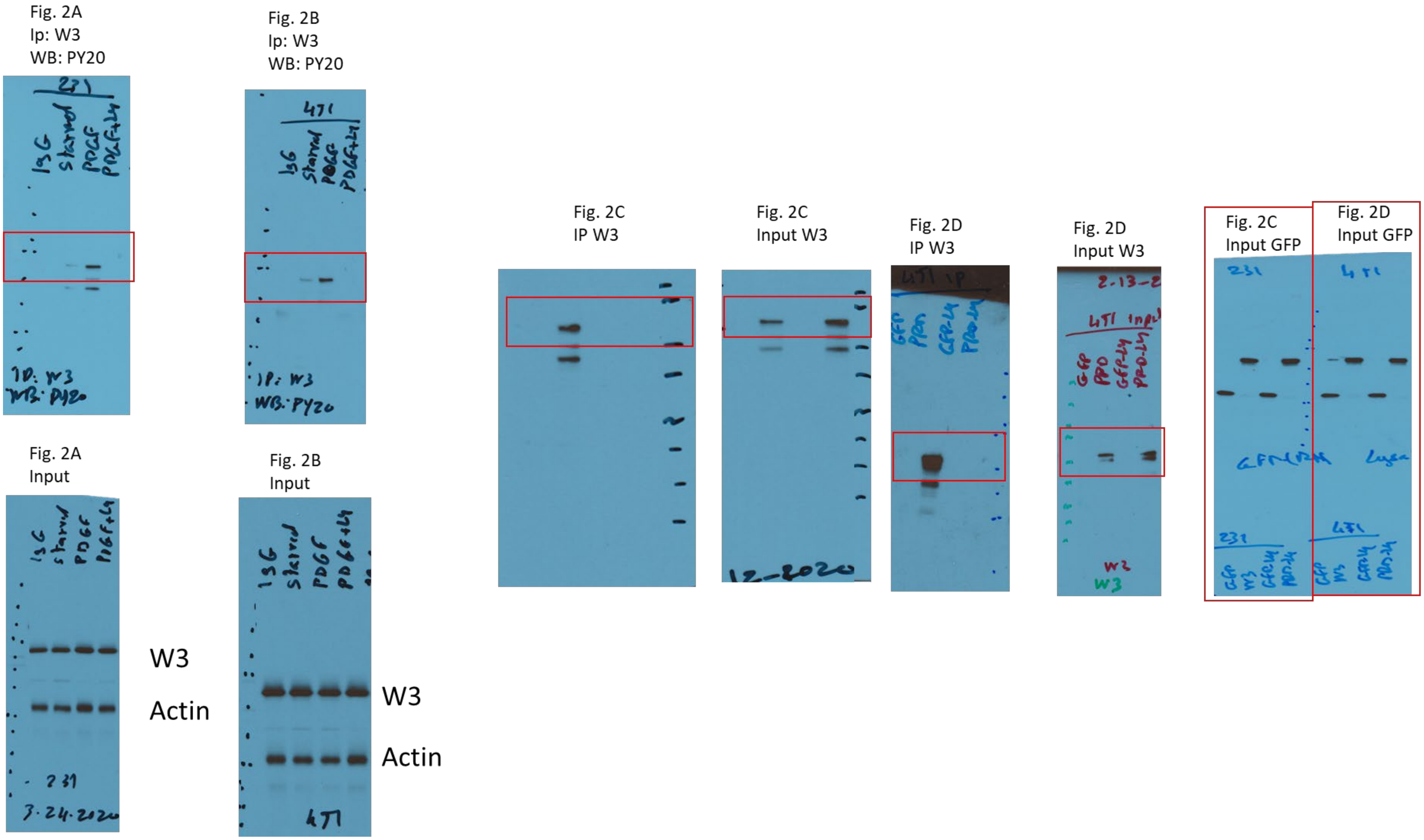
Fig. 2A
Ip: W3
WB: PY20
Fig. 2B
Ip: W3
WB: PY20
Fig. 2C
IP W3
Fig. 2C
Input W3
Fig. 2D
IP W3
Fig. 2D
Input W3
Fig. 2C
Input GFP
Fig. 2D
Input GFP
Fig. 2A
Input
W3
Actin
Fig. 2B
Input
W3
Actin

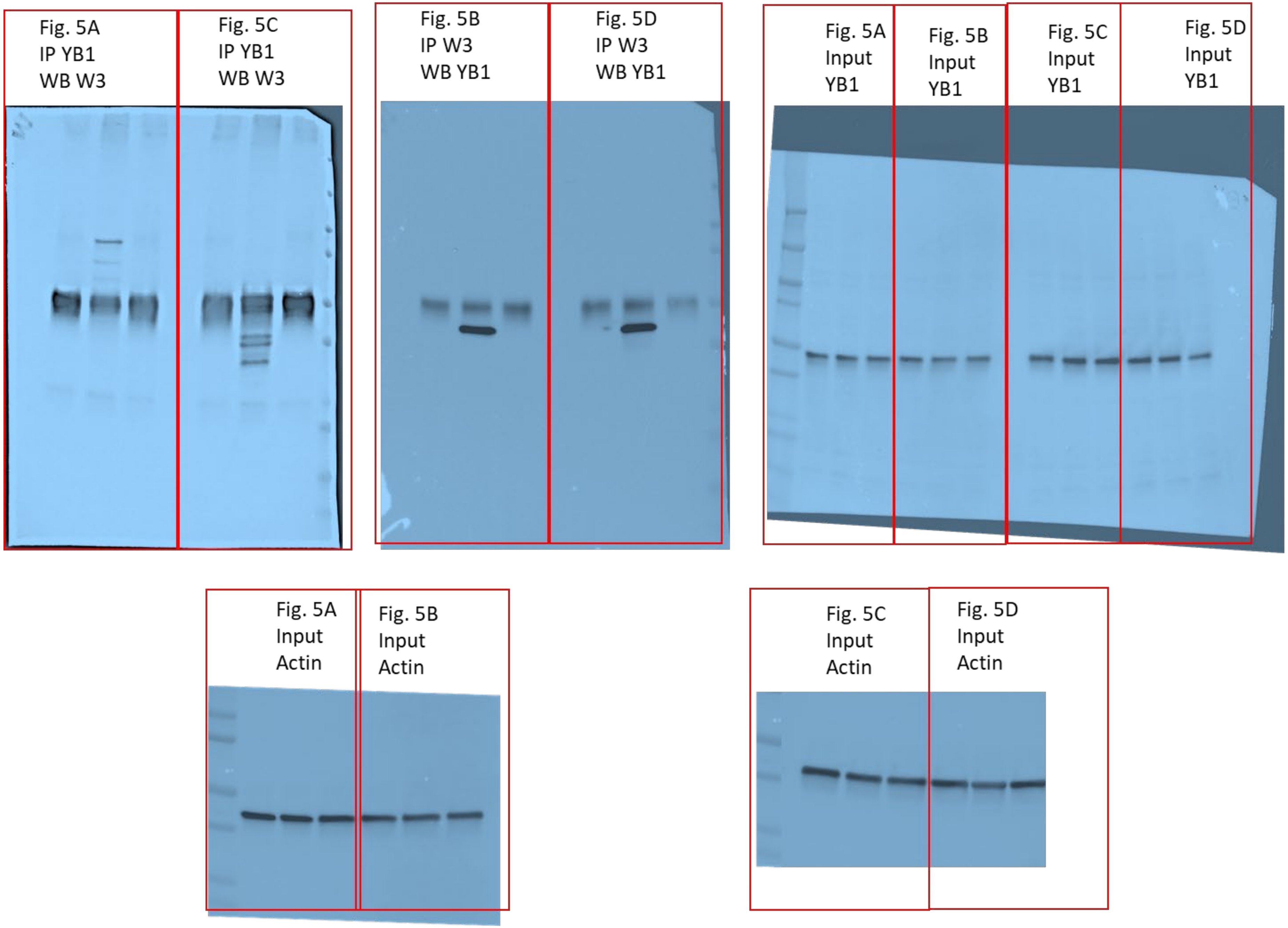
Fig. 5A
IP YB1
WB W3
Fig. 5C
IP YB1
WB W3
Fig. 5B
IP W3
WB YB1
Fig. 5D
IP W3
WB YB1
Fig. 5A
Input
YB1
Fig. 5B
Input
YB1
Fig. 5C
Input
YB1
Fig. 5D
Input
YB1
Fig. 5A
Input
Actin
Fig. 5B
Input
Actin
Fig. 5C
Input
Actin
Fig. 5D
Input
Actin

Figure 7B E-Cadherin

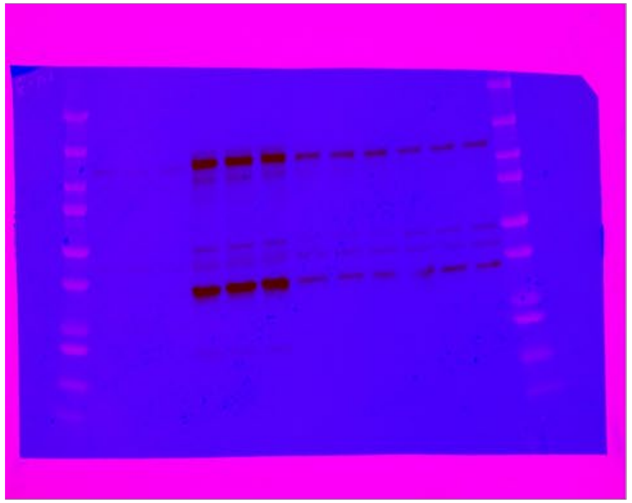

Figure 7B Twist

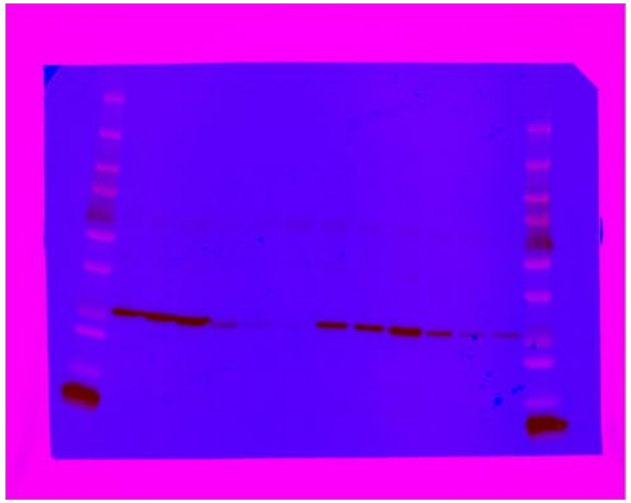

Figure 7B Vimentin

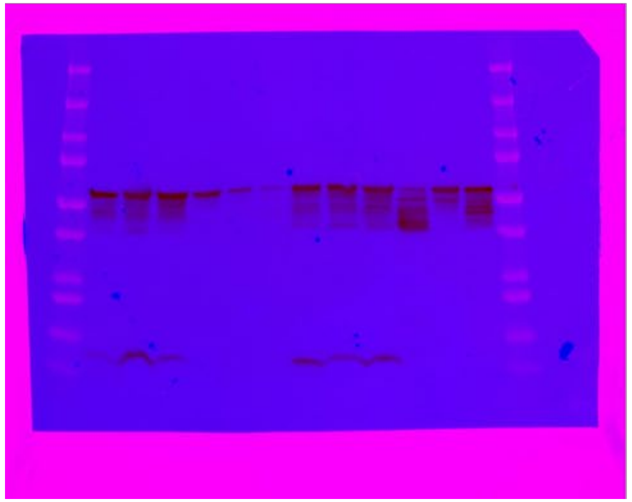

Figure 7B Actin

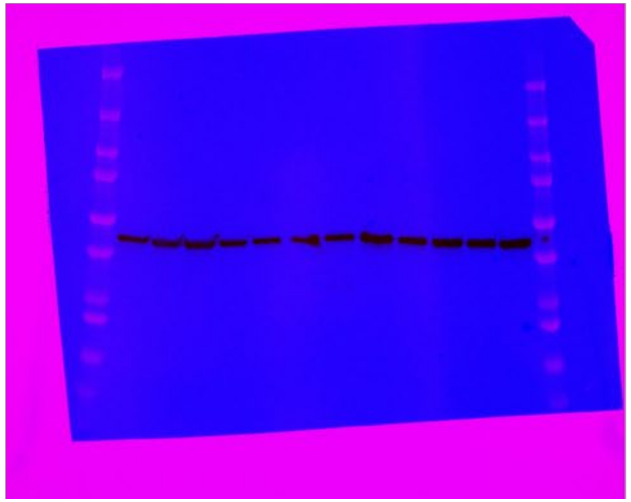

Supplement: Supplementary file 2 — Supplementary Information 2. [file 41598_2021_83479_MOESM2_ESM.pdf]
